# Molecularly Engineered Multifunctional Bridging Layer Derived from Dithiafulavene Capped Spiroxanthene for Stable and Efficient Perovskite Solar Cells

**DOI:** 10.1021/acsami.3c19619

**Published:** 2024-03-26

**Authors:** Afzal Siddiqui, Faranak Sadegh, Kodali Phani Kumar, Priksha Rana, Pankaj Yadav, Daniel Prochowicz, Surya Prakash Singh, Seckin Akin

**Affiliations:** † Department of Polymers and Functional Materials, CSIR-Indian Institute of Chemical Technology (IICT), Uppal Road, Tarnaka, Hyderabad 500007, India; ‡ Academy of Scientific and Innovative Research (AcSIR), Ghaziabad 201002, Uttar Pradesh, India; § Laboratory of Advanced Materials & Photovoltaics (LAMPs), Necmettin Erbakan University, 42090 Konya, Turkey; ∥ Department of Solar Energy, School of Energy Technology, Pandit Deendayal Energy University, Gandhinagar 382007, Gujarat, India; ⊥ Department of Physics, School of Energy Technology, Pandit Deendayal Energy University, Gandhinagar 382007, Gujarat, India; # Institute of Physical Chemistry, Polish Academy of Sciences, 01-224 Warsaw, Poland; ∇ Department of Metallurgical and Materials Engineering, Necmettin Erbakan University, 42090 Konya, Turkey

**Keywords:** molecular engineering, dithiafulvene, bridging layer, uncoordinated Pb^2+^, efficiency and stability, perovskite solar cells

## Abstract

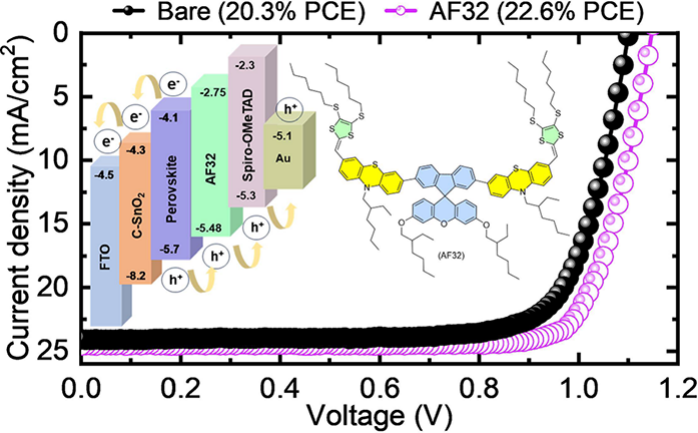

This study introduces a novel approach centered around the design and synthesis of an interfacial passivating layer in perovskite solar cells (PSCs). This architectural innovation is realized through the development of a specialized material, termed dithiafulvene end-capped Spiro[fluorene-9,9′-xanthene], denoted by the acronym AF32. In this design architecture, dithiafulvene is thoughtfully attached to the spiroxanthene fluorene core with phenothiazine as the spacer unit, possessing multiple alkyl chains. AF32 passivates interfacial defects by coordinating the sulfur constituents of the phenothiazine and dithiafulvene frameworks to the uncoordinated Pb^2+^ cations on the surface of the perovskite film, and the alkyl chains construct a hydrophobic environment, preventing moisture from entering the hydrophilic perovskite layer and improving the long-term stability of PSCs. Furthermore, this conductive interlayer facilitates hole transport in PSCs due to its well-aligned molecular orbital levels. Such improvements translated into an enhanced power conversion efficiency (PCE) of 22.6% for the device employing 1.5 mg/mL AF32, and it maintained 85% of its initial PCE after more than 1800 h under ambient conditions [illumination and 45 ± 5% relative humidity (RH)]. This study not only marks progress in photovoltaic technology but also expands our understanding of manipulating interfacial properties for optimized device performance and stability.

## Introduction

Over the past decade, hybrid organic–inorganic perovskites have emerged as prominent semiconducting materials within photovoltaic devices, owing to their remarkable optoelectronic attributes. These materials possess favorable and tunable band gaps conducive to efficient sunlight absorption, elevated optical absorption coefficients, extended charge-carrier lifetimes, considerable diffusion lengths, and facile processing manufacturing.^1^ Hybrid organic–inorganic perovskite solar cells (PSCs) have achieved noteworthy advancements in efficiency within recent years and achieved a pinnacle power conversion efficiency (PCE) exceeding 26%, as validated by the National Renewable Energy Laboratory (NREL).^2^ However, despite this surge in efficiency, the concurrent attainment of prolonged device stability and optimal performance remains a formidable challenge. This challenge arises from the inherent susceptibility of PSCs to degradation upon exposure to diverse factors, including moisture, light, heat, and oxygen, thereby constraining their feasibility for commercial deployment. In addition, the solution-processed perovskites undergo rapid crystallization and a series of ionic defects on the surface and the grain boundaries of the perovskite crystal lattice, such as uncoordinated Pb^2+^ cation.^3^ These defects not only cause nonradiative charge recombination and hysteresis in the current–voltage (*I–V*) characteristics of PSCs but also make the perovskite layer prone to humidity, oxygen, and heat, resulting in irreversible degradation of the perovskite.^4,5^ Hence, it is of great significance to design appropriate interfacial layers to construct highly efficient and stable PSCs.

Interfacial engineering is one of the most promising approaches to respond to interfacial defects, which involves the incorporation of the passivating material or an interfacial layer between the perovskite and the charge transport layer.^6^ An ideal passivating layer serves multiple functions as it not only imparts stability to the system by interacting with the perovskite layer via various electrostatic interactions but also improves charge transportation by having well-aligned energy levels with charge transport as well as the perovskite layers, thereby increasing the efficiency of the PSC.^7−9^

Several approaches to address defect passivation in PSCs have been discussed and reported, such as the use of organic ammonium salts,^10^ ionic liquids,^11^ self-assembled molecules,^12^ polymers,^13^ and fullerene derivatives.^14,15^ In addition to that, molecules with specific functional groups such as amino, carboxyl, sulfonate, cyano, carbonyl, fluorine, thiophene, and pyridine have been used as passivating materials in PSCs due to their interaction tendency with uncoordinated Pb^2+^ present on the surface of the perovskite layer.^16−23^ These passivation agents are mostly insulators that passivate the traps, causing charge recombination and providing environmental stability to the PSCs. Being electrically insulating in nature, these passivation agents do not contribute toward the charge transfer across the interfacial layer and can result in high electrical resistance in the device. Therefore, for the construction of stable and efficient PSCs, it is crucial to develop a versatile interfacial layer that not only passivates the interfacial defects but also promotes charge transportation. In the past few years, π-spacer engineering has emerged as a new strategy to alter the properties of small molecules by connecting them to various donor or acceptor fragments, long alkyl chains, and functional groups to modulate various properties such as absorption, band gap, hole mobility, and performance.^24−26^ In this regard, different research groups developed sulfur-rich semiconducting materials and achieved enhanced PCE. For instance, Zhong and co-workers reported a two-dimensional sulfur-containing pyrene-based interfacial layer which increased the PCE from 20.4 to 22.3%.^27^ Recently, Wu and co-workers utilized a methylthiophene-based D-π-D interfacial material to enhance the stability of the PSC and achieved an efficiency of 20.11%.^28^ Gao and co-workers developed a series of D-π-A porphyrin molecules having cyanoacrylic acid to passivate the interfacial defects and achieved an enhanced PCE of 22.37%.^29^

**Scheme 1:**
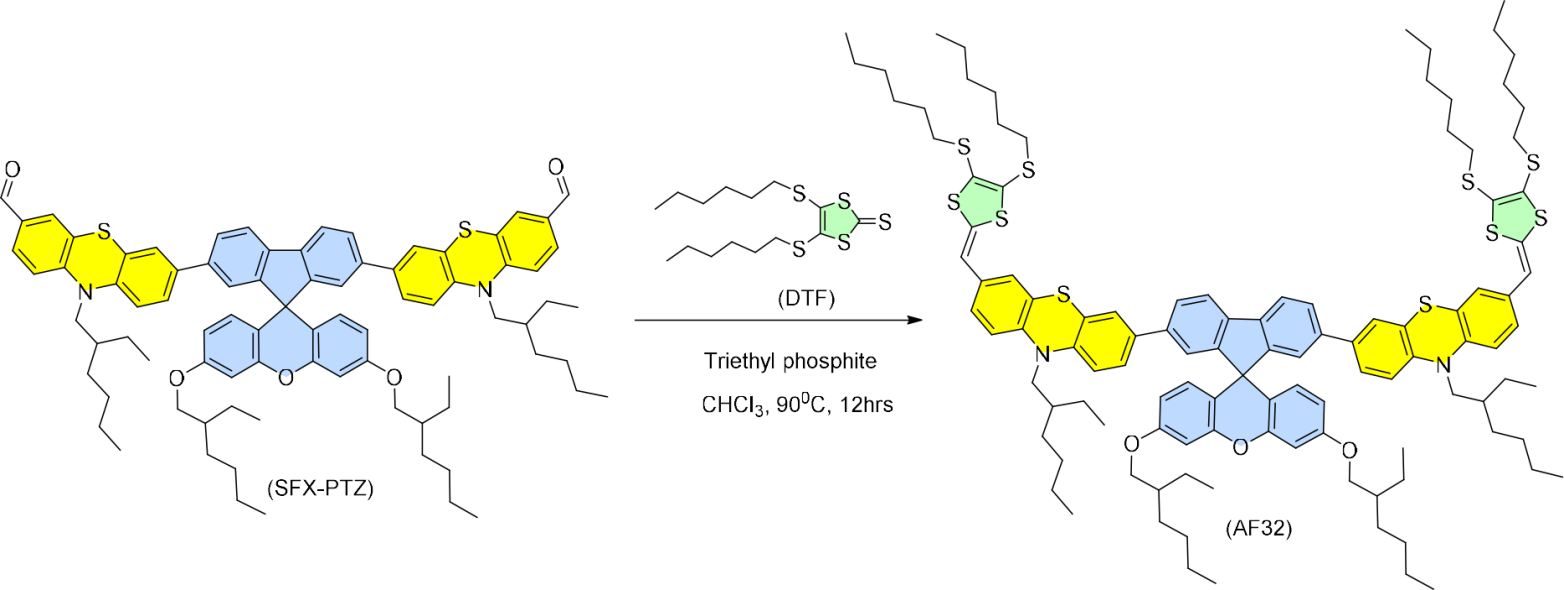
Synthetic Scheme of AF32

Herein, we have designed and synthesized a conjugated donor-π-acceptor system (D-π-A), AF32, and used it as an interfacial material featuring Spiro[fluorene-9,9′-xanthene] (SFX) as the middle core and phenothiazine (PTZ) as the π-spacer unit end-capped by dithiafulavene (DTF), as demonstrated in Scheme 1. SFX, a substitute of the spiro-bifluorene core in state of art spiro-OMeTAD, has been extensively used as a core molecule in the hole transporting materials (HTMs) in PSCs because of its low-cost, one-pot facile synthesis, nonplanarity, and its ability to form a good homogeneous layer facilitating the charge transfer across the layer.^30−34^ However, to the best of our knowledge, molecules with an SFX core have not been used as interfacial material. Based on these properties, SFX was chosen as the central core molecule. Our further approach was to introduce sulfur components/constituents in the molecule because of their strong ability to bind with the uncoordinated Pb^2+^ cations present on the perovskite surface.^28^ DTF appeared to be the best candidate to serve this purpose as it not only provides multiple sulfur sites but also acts as a strong electron donor and modulates the optoelectronic properties of the material.^35^ Due to its unique charge transfer and redox characteristics, DTF has numerous applications in photonic devices and organic conductive materials; however, its application in the design of passivation agents has yet to be explored.^36,37^ Its nonplanar conformation and electron-rich nitrogen and sulfur atoms make PTZ a promising material for photovoltaic applications. We selected PTZ as the π-spacer as it provides the necessary sulfur atoms for defect passivation and as it can be further functionalized by alkyl chains on its nitrogen atom. Furthermore, the incorporation of multiple long alkyl chains within AF32 serves the purpose of instilling a hydrophobic nature into the interfacial layer. This hydrophobic attribute plays a pivotal role in preventing moisture ingress into the hydrophilic perovskite layer. The photovoltaic results demonstrate that the designed AF32 effectively passivates the uncoordinated Pb^2+^ defects on the hybrid perovskite layer, resulting in an enhanced PCE of 22.6% in the case of the modified champion device compared to the control device without the interfacial layer with a PCE of 20.3%. Most strikingly, the device based on AF32-1.5 (1.5 mg/mL in concentration) as an interfacial layer was found to maintain 85% of its initial PCE over 1800 h under ambient conditions (illuminated at 45 ± 5% RH), whereas the PCE of the control device was significantly reduced to 35% of its initial value over the same time frame.

## Materials and Methods

### Characterizations of AF32 Molecules

All precursor materials were purchased directly from commercial sources and used as such without any further purification unless otherwise specified. Solvents used for the reaction were dried under vacuum by following the standard procedure. All the reactions were carried out under inert conditions in the presence of N_2_. Purification of crude compounds was performed by column chromatography using 100–200 mesh silica. ^1^H and ^13^C NMR were recorded at room temperature using BRUKER Avance 101, 300, 400, and 500 MHz instruments. Spectra was recorded by dissolving compounds in deuterated chloroform and using tetramethylsilane (TMS) as the internal standard and represented on a parts per million (ppm) scale. Abbreviations s, d, t, dd, and m correspond to singlet, doublet, triplet, doublet of doublet, and multiplet, respectively. High-resolution mass spectra were measured on a Shimadzu LCMS-2010 EV model with an ESI probe, and the final compounds were also characterized by using MALDI-TOF. UV–vis analysis was carried out using a SHIMADZU UV-1800 instrument. Solvents used for absorption and cyclic voltammetry analyses are of HPLC grade purchased from Finar. Cyclic voltammetry was performed on CH-Instruments with a three-electrode system consisting of a Ag/AgCl reference electrode, a working electrode, and a platinum wire counter-electrode. The redox electronic potentials of the dyes were measured in chloroform (CHCl_3_) containing 0.1 M Bu_4_N HClO_4_ at a scan rate of 100 mVs^–1^.

### Fabrication of Films and Devices

The FTO substrates were etched with zinc powder and HCl (4 M) to obtain the required electrode pattern and consecutively cleaned using 2% Hellmanex aqueous solution, deionized water, acetone, and ethanol by sonicating for 15 min in each solvent. After drying the substrates, they were treated with ultraviolet ozone (UV–O_3_) for 20 min for further cleaning of organic residuals; 20–30 nm thick compact SnO_2_ (c-SnO_2_) film was deposited on top of FTO substrates via spin-coating of a diluted solution of colloidal dispersion (volume ratio of water:colloidal SnO_2_ solution, 5.5:1) at 3000 rpm for 30 s. Subsequently, the substrates were sintered at 180 °C for 30 min in air. Triple cation perovskite solution having composition of [Cs_0.05_(FA_0.95_MA_0.05_)_0.95_Pb(I_0.95_Br_0.05_)_3_] was prepared with CsI, FAI, MABr, PbI_2_, and PbBr_2_ precursors in DMSO:DMF (1:4, v:v) solvents under mild heating condition at ∼70 °C to assist dissolving. The solution of perovskite was spin-coated at 1000 rpm for 10 s and then 6000 rpm for 20 s. During the second step, chlorobenzene as antisolvent was poured on the spinning perovskite surface 10 s before the end of time. Then, all substrates were transferred to the hot plate immediately and annealed at 100 °C for 45–50 min. For AF32 treatment, different concentrations of AF32 (0.5, 1.0, 1.5, 2.0, 5.0 mg/mL) were dissolved in anhydrous toluene without heat treatment and spin-coated on the surface of the perovskite at 5000 rpm for 20 s. Subsequently, HTL solution (70 mM spiro-OMeTAD in 1 mL of chlorobenzene including 36 μL of tBP and 18 μL of Li-TFSI solution (520 mg Li-TFSI in 1 mL acetonitrile)) was spin-coated with 4000 rpm for 30 s. Finally, 80 nm of the gold electrode was deposited with the thermal evaporator.

### Characterizations of Films and Devices

The crystallinity of the pristine and modified perovskite films was evaluated by X-ray diffraction (XRD) (D8 Advance, Bruker). X-ray photoelectron spectroscopy (XPS) measurements were performed by using a scanning XPS microprobe (Thermo Scientific K-Alpha). The surface morphology of the perovskite films was characterized by field emission scanning electron microscopy (FE-SEM) (S*-*5500, Hitachi) and atomic force microscopy (Park System) with Kelvin probe attachment. The UV–vis absorption spectra were recorded with a spectrophotometer (UV*-*1800, Shimadzu). The contact angle measurements were assessed by a drop-shape analyzer (KRUSS, DSA100). The photoluminescence (PL) measurements were carried out by Hitachi fluorescence spectrophotometer F-7100. The time-resolved PL (TRPL) spectra were measured by Edinburgh Instruments FLSP920. The TRPL spectra were fitted with a double-exponential decay function. The space charge limited current (SCLC) measurements were performed by collecting the *I–V* characteristics of devices in the configuration of FTO/PTAA/perovskite with and without AF32/spiro-OMeTAD/Au. EIS measurements of PSCs were performed by using Ivium potentiostat under the dark condition at a constant bias of 1.0 V in the frequency range from 1 MHz to 1 Hz. The photovoltaic performance of the fabricated solar cells was recorded using a 450 W xenon light source (Oriel) under irradiation of 100 mW cm^–2^ at an AM of 1.5 G under ambient conditions. The light intensity was calibrated with a Si photodiode. The *J–V* characteristics of all devices were measured by masking the active area with a metal mask of area 0.16 cm^2^ and applying an external potential to the cell while recording the generated photocurrent with a digital source meter (Keithley 2400) without applying any device preconditioning such as prolonged light soaking or forward voltage bias before starting the measurement. external quantum efficiency (EQE) spectra of the corresponding devices were recorded as a function of wavelength (350–850 nm) with a 150 W xenon arc lamp source (QEX10, PV Measurements). For stability measurements of devices, the current density–voltage (*J*–*V*) curves of cells stored without encapsulation under ambient conditions were periodically recorded under light at room temperature.

## Results and Discussion

**Figure 1:**
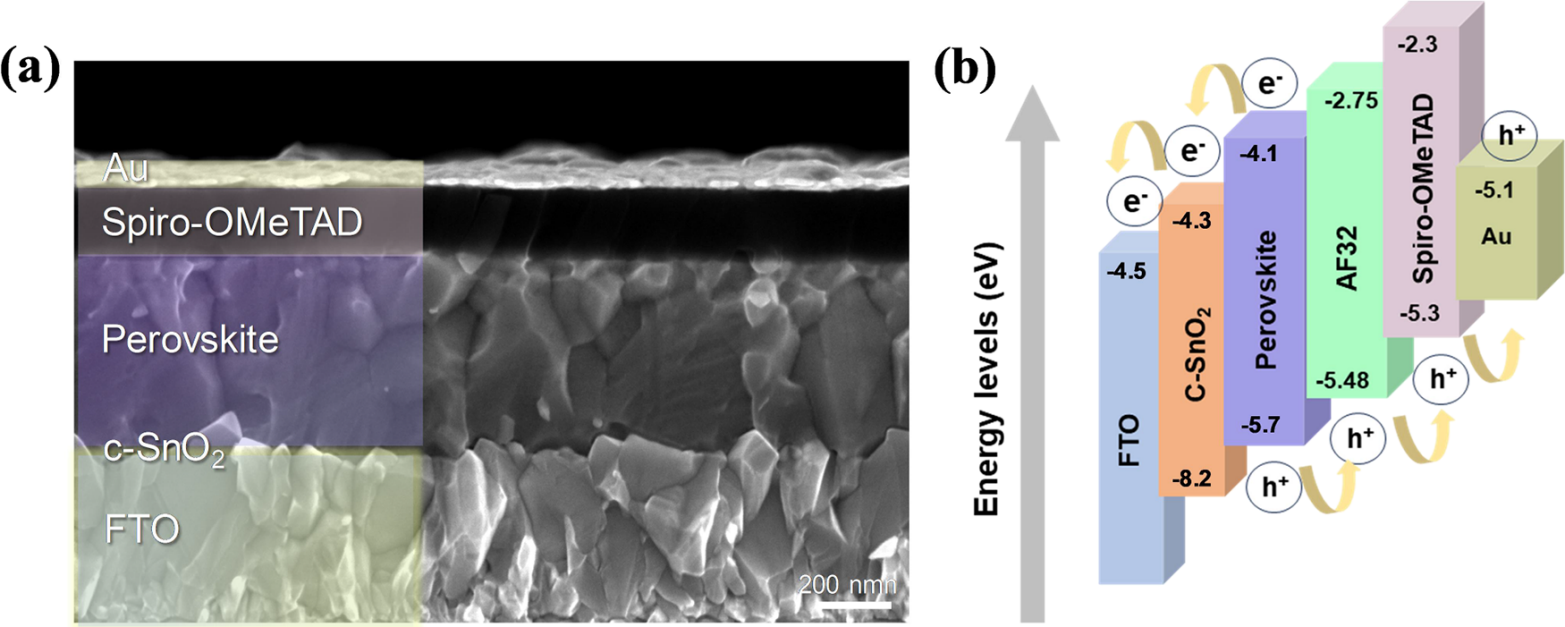
(a) Cross-sectional SEM image of the corresponding device. (b) Schematic of the energy alignment of the full device demonstrating the HOMO and LUMO levels.

The synthetic route for the preparation of AF32 is represented in Figure S1 where the key step involves the Pd(PPh_3_)_4_-mediated classical Suzuki coupling between the boronic ester (3) and the bromo phenothiazine carbaldehyde to produce 4. The final step involves the Horner–Wittig condensation reaction between the obtained aldehyde 4 and DTF to produce the D-π-A unit in the final molecule AF32. The detailed synthetic procedure (Supplementary Note 1) and all the characteristic data (^1^H NMR, ^13^C NMR, and MALDI-TOF spectra of AF32) are provided in the Supporting Information. To understand the absorption properties of the AF32 molecule, the UV–vis spectrum was recorded in dichloromethane solvent (Figure S2a). AF32 exhibited a broad absorption range of 300–450 nm, spanning the UV to visible regions. The strong intensity peak at 394 nm, with a molar extinction coefficient of 44730 M^–1^ cm^–1^ (Figure S2b) is attributed to the intramolecular charge transfer (ICT) from the donors to the acceptor in the molecule. However, another absorption peak at 330 nm arises from the localized π–π* transitions present in this conjugated backbone. To understand the redox properties of the AF32 molecule, cyclic voltammetry (CV) and differential pulse voltammetry (DPV) were conducted in anhydrous dichloromethane solvent under nitrogen at room temperature; 0.1 M tetrabutylammonium perchlorate (TBAF), ferrocene/ferrocenium (Fc/Fc^+^), platinum wire, and Ag/Ag^+^ were used as supporting electrolyte, redox couple, counter electrode, and reference electrode, respectively. CV and DPV plots have been depicted in Figure S2c,d and the resultant data were summarized in Table S1. The highest occupied molecular orbital (HOMO) energy level of AF32 was calculated from an empirical formula *E*_HOMO_ = −e [*E*_*ox*_ + 4.80 – *E*_(Fc/Fc+)_] as −5.48 eV. The lowest unoccupied molecular orbital (LUMO) energy level is determined using the equation *E*_LUMO_ = *E*_HOMO_ + *E*_0–0_ as −2.75 eV. The HOMO–LUMO energy levels are well-aligned with Spiro-OMeTAD and the perovskite layers to facilitate efficient hole extraction. The cross-sectional scanning electron microscopy (SEM) view and schematic of the device architecture employed in this study and energy alignment of the full device demonstrating the HOMO and LUMO levels can be seen in Figure 1.

**Figure 2:**
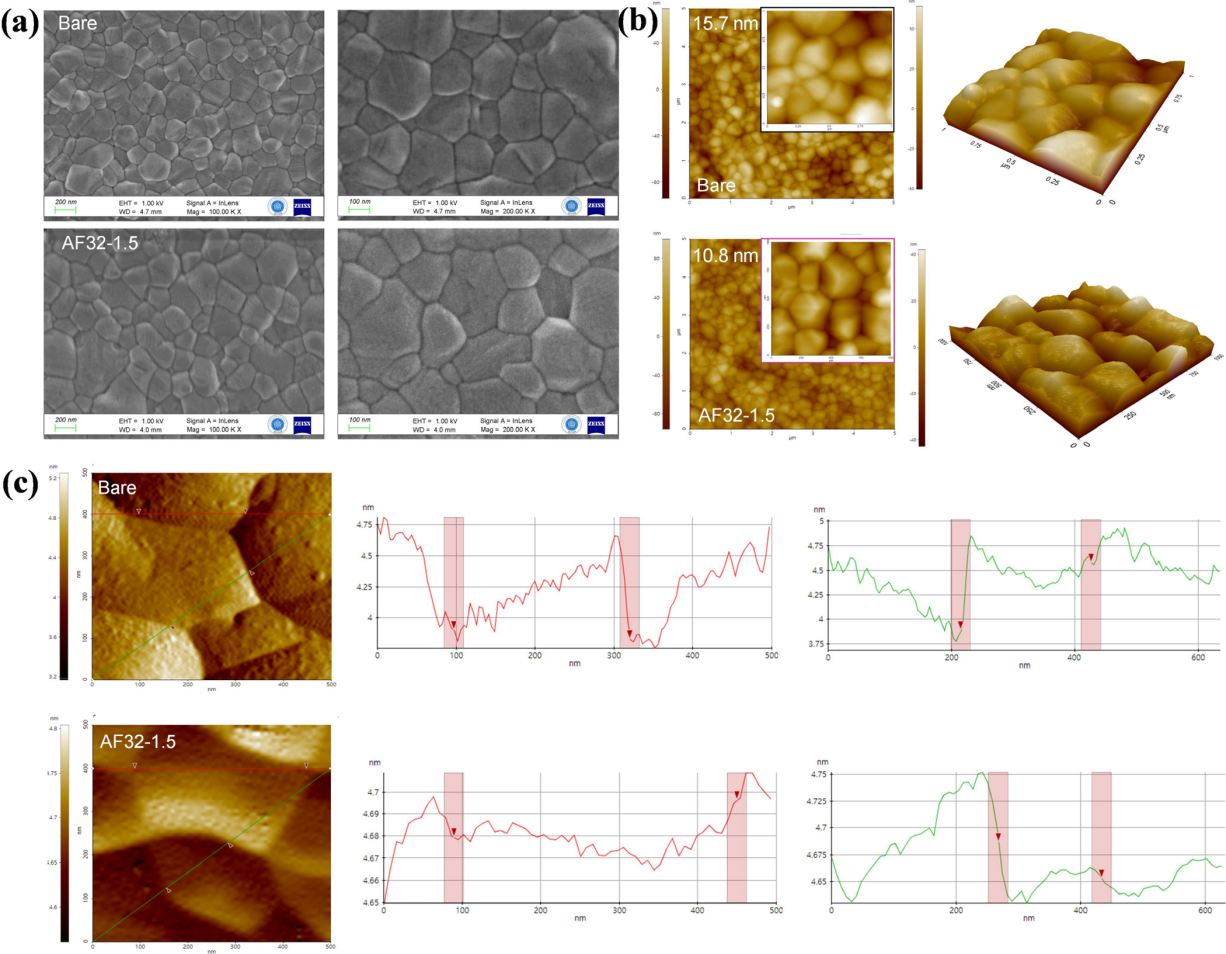
(a) Top-view SEM micrographs at different magnifications of perovskite films without and with AF32-1.5. (b) Top-view AFM images and (c) AFM height maps of perovskite films without and with AF32-1.5.

The morphology of the control and AF32 passivated perovskite layers was investigated by using SEM top-view images (Figures 2a and S3). Grain size histograms demonstrate that there is an observable change in the grain size of the perovskite films after the insertion of the AF32 (Figure S4). This improvement can be explained by the passivation phenomenon of the AF32 molecule, which occurs due to the process of recrystallization. Additionally, following the passivation of the perovskite surface with AF32, the resultant perovskite film exhibits robustly formed and densely arranged grains. This suggests that AF32 has the capability of being deposited at the surface or boundaries of the grain in the perovskite film, effectively filling in the defects of the film. This development leads to the creation of a high-quality perovskite film that demonstrates a surface passivation effect on defects, thereby reducing charge recombination. This favorable characteristic aids in facilitating the movement of charges across layers. Moreover, we recorded top-view atomic force microscopy (AFM) images of the corresponding films. As can be seen in Figures 2b and S5, AFM studies reflected that the perovskite film without AF32 exhibits a rough surface morphology with root-mean-square (RMS) of 15.7 nm. Despite the anomaly where the RMS of AF32-1.0 is 14.4 nm, larger than that of AF32-0.5 (13.3 nm), the overall trend indicates a decreasing RMS with an increasing AF32 concentration. Upon insertion of the AF32 on top of the perovskite film, the RMS was decreased to 5.9 nm for AF32-5.0, which signifies that the passivation introduces uniformity to the surface of the perovskite layer in terms of spatial and height aspects, indicating that AF32 is deposited at the grain boundary and reduces the height difference between the grain surface and grain boundary (Figure 2c).^38^ The smoothness in the perovskite layer improves hole transport across the layer by providing better physical contact between the perovskite layer and the hole transport layer (HTL).

**Figure 3:**
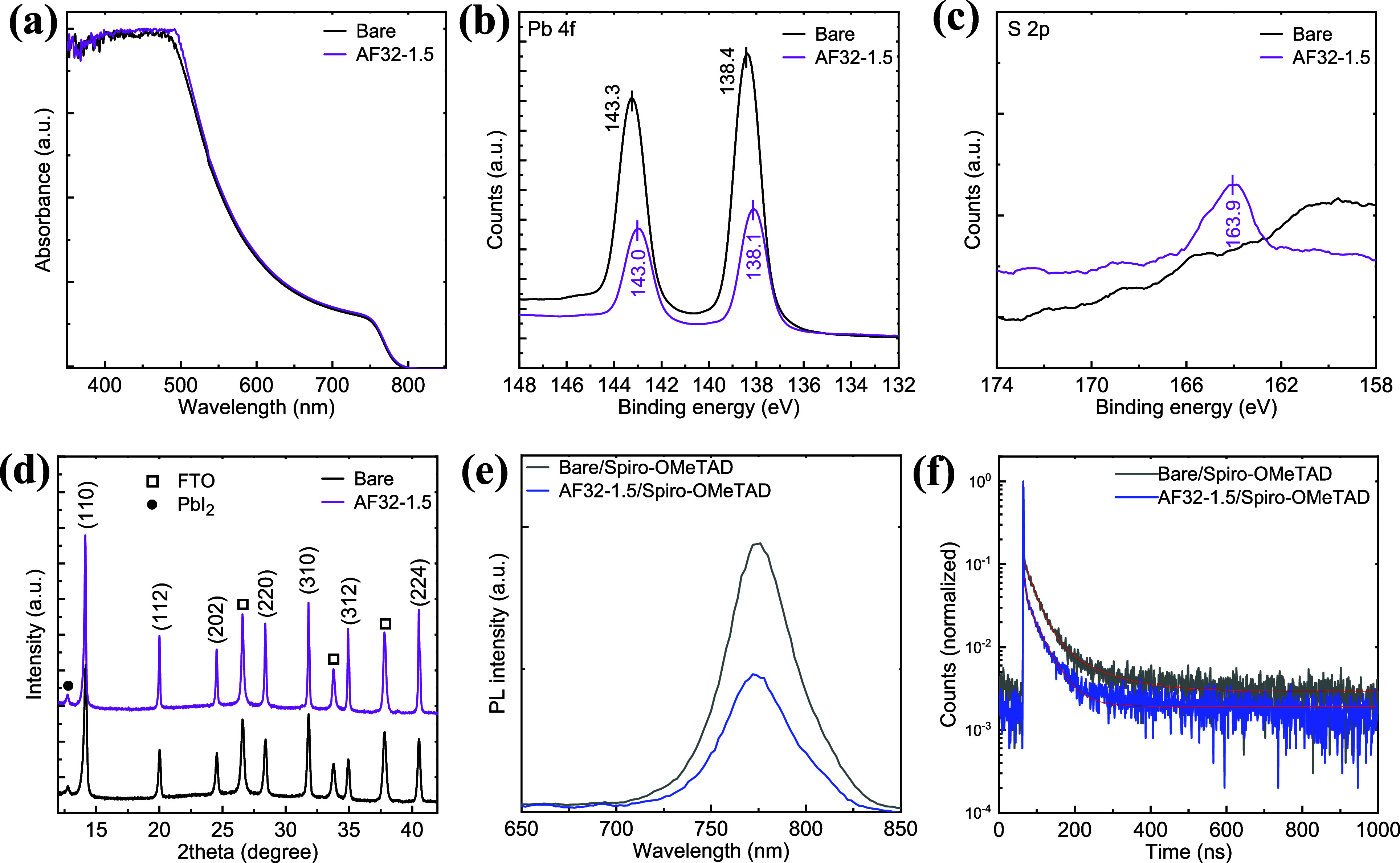
(a) UV–visible absorption spectra of the perovskite films without and with AF32. High-resolution (b) Pb 4f and (c) S 2p core level XPS spectra of perovskite films. (d) XRD patterns of the perovskite films without and with AF32. (e) PL and (f) TRPL spectra of the perovskite films in the presence of the spiro-OMeTAD layer.

The ultraviolet–visible (UV–vis) spectra of these perovskite films were analyzed to investigate the influence of AF32 on light absorption (Figures 3a and S6). Upon comparison to the pristine perovskite, similar absorbance patterns were observed in films containing different concentrations of AF32. Remarkably, the passivation layer, due to its extremely thin profile, exerts minimal influence on light harvesting. To study the interaction between AF32 and the perovskite layer, X-ray photoelectron spectroscopy (XPS) was conducted. As shown in Figure 3b, the binding energy of the Pb core level in the pristine perovskite film is located at 143.3 and 138.4 eV corresponding to 4f_5/2_ and Pb 4f_7/2_, respectively. Upon passivation with AF32, the peaks of Pb 4f_5/2_ and Pb 4f_7/2_ were shifted to 143.0 and 138.1 eV, respectively, with a 0.3 eV shift to the lower binding energy values. This shows a change in electron density on the Pb nucleus caused by the strong coordination of the AF32 layer with the uncoordinated Pb^2+^ cations at the surface of the perovskite layer. A similar shift is also observable in other spectra provided in Figure S7. As given in Figure 3c, the small peak observed in the sulfur (S) 2p spectrum, in line with the value as verified in the literature,^39^ signifies the existence of the AF32 molecule atop the perovskite film, suggesting that the S atoms within the AF32 molecule have the potential to coordinate with the uncoordinated Pb^2+^ ions on the perovskite surface.^27,28^ This coordination capability enables them to passivate the halide vacancies, considered primary defects, thereby enhancing the performance of PSCs due to the suppressed nonradiative recombination.

X-ray diffraction (XRD) experiments were carried out to better understand the effect of passivation with AF32 on the crystallinity of the perovskite layer. Figure 3d shows the XRD patterns of the pristine perovskite film and the passivated perovskite film with AF32. As calculated from the pseudo-Voigt distribution (not shown here), the full width at half-maximum (fwhm) values of the (110) peak are 0.25° and 0.15° for control and passivated perovskite films, respectively. It was observed that the crystallinity improved after passivation, as evidenced by the improved intensity of the peak (110), which is again in agreement with the enlarged grains in the SEM image of the passivated layer. The residual PbI_2_, which has the characteristic peak at 2θ = 12.8°, was observed for both films. Furthermore, the XRD pattern of the passivated perovskite film with AF32 is similar to that of the pristine perovskite layer, with no discernible peak shift, indicating that the crystal structure of the perovskite layer remains unaffected after passivation. Hence, the passivation with AF32 improves the crystalline nature of the perovskite layer, which can be correlated to the AF32, which stimulates crystal development in the perovskite layer.^40,41^

We also aimed to investigate the charge transfer dynamics occurring at the interface of the perovskite/spiro-OMeTAD through the utilization of photoluminescence (PL) and time-resolved photoluminescence (TRPL) analyses in the presence and absence of AF32. The peak intensity was found to be reduced in the presence of the AF32 layer, indicating a superior hole extraction from the perovskite film to spiro-OMeTAD (Figure 3e). The more efficient PL quenching was discovered when the AF32 layer was introduced between the perovskite and HTL, suggesting that the interfacial conducting passivating layer enhances hole transfer/extraction across the layers.^42^ As shown in Figure 3f and summarized in Table S2, TRPL shows a faster average decay lifetime (τ_ave_) with the sample of perovskite/AF32/spiro-OMeTAD (29.6 ns) as compared to the control film (55.4 ns) that shows longer hole transfer times. This suggests that AF32 is somewhat effective in promoting hole extraction if we assume that other factors do not contribute to the recombination process at the interface. Specifically, the decline in τ_1_ (28.2–4.9 ns) suggests that the AF32 modification improved carrier extraction efficiency, likely due to the enhanced uniformity and better alignment of energy levels between the HTL and perovskite. This enhancement may result in a potential slight increase in the short-circuit photocurrent density (*J*_SC_). On the other hand, the decrease in τ_2_ (107.6–40.2 ns) indicates a lowering of defect density within the perovskite. This reduction could be linked to the passivation of surface defects, potentially leading to an increase in both the open-circuit voltage (*V*_OC_) and fill factor (FF).

**Figure 4:**
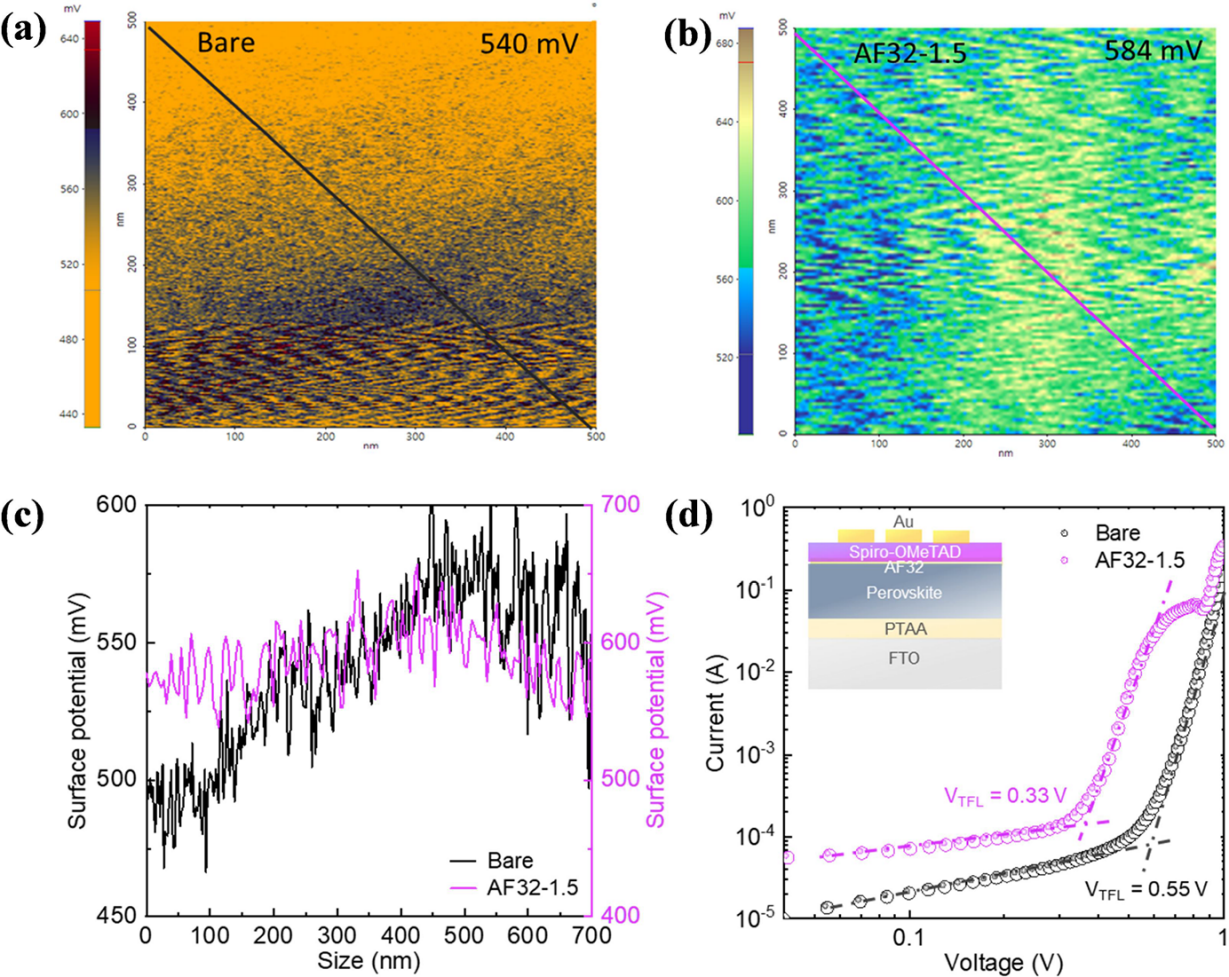
KPFM images of (a) bare and (b) AF32-modified perovskite films. (c) Potential difference curves of the perovskite films with and without AF32. (d) Dark *J–V* characteristics of SCLC measurement for hole-only devices based on the perovskite films without and with AF32. The inset shows the corresponding device configuration.

Kelvin probe force microscopy (KPFM) was employed for a detailed assessment of the surface potential across the various perovskite films. Figure 4a,b presents the KPFM images, depicting a cross scan from the top-left to the bottom-right. This scan traversed through both the grain interior and the grain boundary successively. Defects at deep levels tend to possess low formation energies consequently, they are prone to formation at grain boundaries and/or the surface of perovskite films, significantly influencing the surface potential variations.^43^ As indicated in Figure 4c, the KPFM outcomes revealed a more uniform distribution of surface potential with reduced fluctuations in the AF32-modified perovskite film. This finding strongly implies a substantial reduction in defect states at grain boundaries and the film surface. Moreover, the trap density (*n*_trap_) of the perovskite film was assessed using the space-charge-limited current (SCLC) technique in the configuration of FTO/PTAA/perovskite/with and without AF32/spiro-OMeTAD/Au. Figure 4d represents the dark *J–V* curves of the corresponding devices where *V*_TFL_ signifies the trap-filled limit voltage which is directly proportional to the trap density.^44^ Compared to the control device, the AF32-modified device demonstrated reduced *V*_TFL_ (0.55–0.33 V) and *n*_trap_ (7.8 × 10^15^–4.7 × 10^15^ cm^–3^), once again confirming the efficacy of AF32 passivation in mitigating the prominent defects within perovskites.^45^ These findings are consistent with the PL measurements and contribute significantly to elevated *V*_OC_ values.

**Figure 5:**
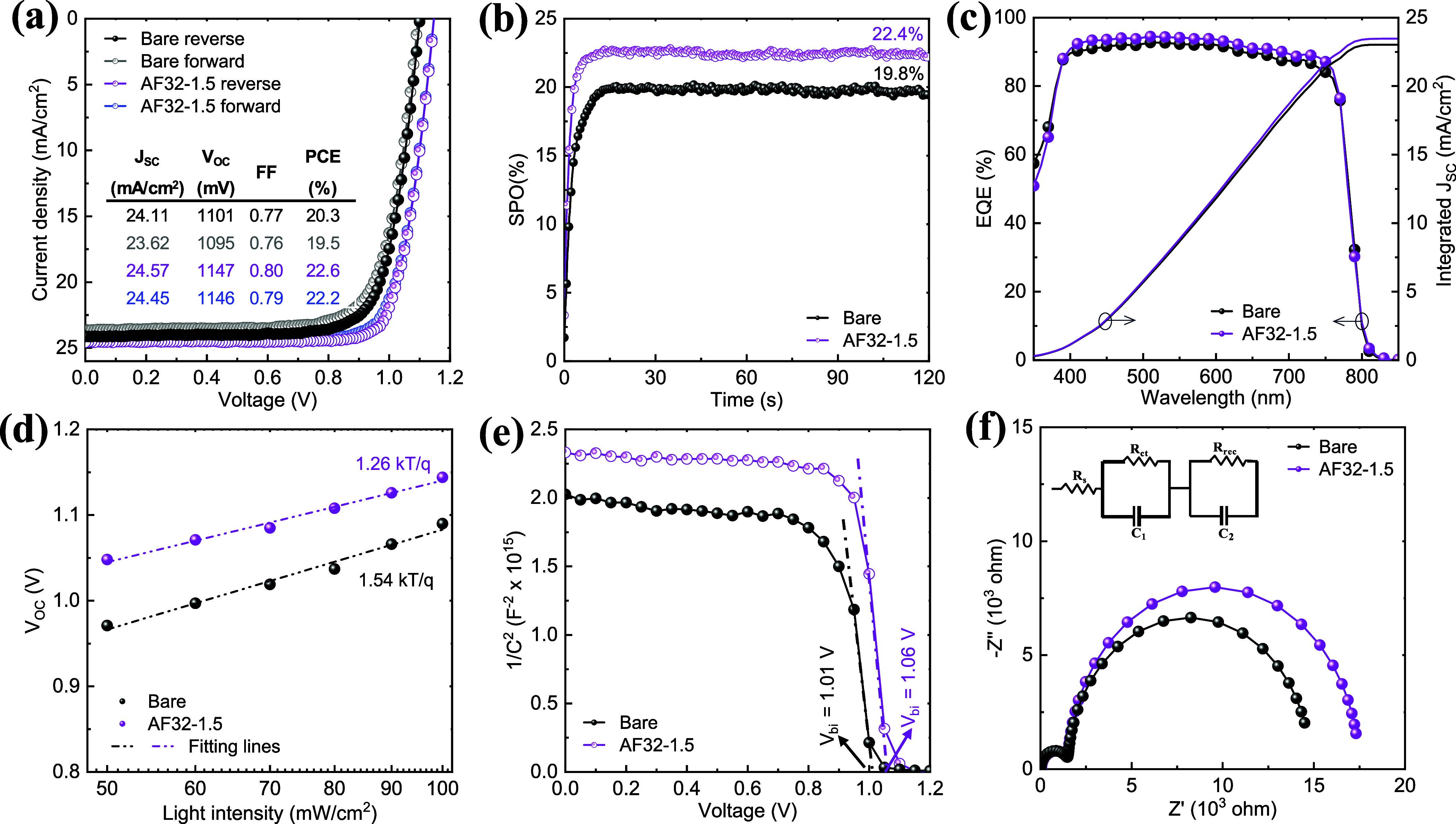
(a) *J–V* curves in reverse and forward scans, (b) steady-state output curves, (c) EQE spectra, (d) light intensity dependence of the *V*_OC_, (e) M–S plots, and (f) EIS curves of devices with and without AF32.

To understand the impact of the insertion of AF32 on the photovoltaic properties, PSCs were fabricated in FTO/c-SnO_2_/perovskite/AF32/spiro-OMeTAD/Au structure imploying various concentrations of AF32 (0, 0.5, 1, 1.5, 2, and 5 mg/mL). As depicted in Figure S8, the best performance was achieved by the devices employing 1.5 mg/mL of AF32, with enhanced photovoltaic (PV) parameters, especially in terms of the *V*_OC_ and FF, which led to a considerable rise in overall PCE. The *J–V* curves of the fabricated champion devices with and without AF32 interlayer, in reverse and forward scans are depicted in Figure 5a, and PV parameters are gathered in Table 1. The control device yielded a power conversion efficiency (PCE) of 20.3% with a *J*_SC_ of 24.11 mA cm^–2^, a *V*_OC_ of 1.10 V, and an FF of 77%. The insertion of the AF32 resulted in a remarkable PCE of 22.6% with a *J*_SC_ of 24.57 mA cm^–2^, a *V*_OC_ of 1.15 V, and a notable FF of 80%. A significant increase in FF and *V*_OC_ can be explained by the decrease in defect states at the interface resulting in suppressed charge recombination processes and energy level alignment between perovskite and HTL which can facilitate charge transportation/extraction.^46^ As demonstrated in Figure S9, the average PCE values from 10 to 20 independent devices consistently demonstrated high reliability, measuring at 19.6 ± 0.3% for control devices and 21.9 ± 0.2% for passivated devices. Additionally, the modified device displayed a negligible hysteresis index (HI) of 1.8% (HI of 3.9% for the control device) when scanned in the forward and reverse directions. The observed hysteresis may be attributed to the instability induced by electrical bias at the perovskite/spiro-OMeTAD interface, which is closely associated with the electrochemical reactivity of the perovskite layer. The AF32 serves to efficiently passivate the surface of the 3D perovskite and enhances the energy band structure at the interface, concurrently inhibiting halide ion migration. As a result, the AF32 layer effectively mitigates the hysteresis effect observed in the devices. As illustrated in Figure 5b, the champion cell demonstrated a steady-state power output (SPO) measured at its maximum power point (MPP), achieving a stabilized PCE of 19.8 and 22.4% for control and passivated devices, respectively. This closely matches the device performance obtained from the *J–V* characteristics. Figure 5c represents the EQE spectra of the control and modified devices. Compared to the control device, the passivated device shows an enhanced photocurrent response over the whole range of wavelengths. The corresponding integral *J*_SC_ values for control and modified devices were found as 23.04 and 23.48 mA cm^–2^, respectively, which is in agreement with the *J*_SC_ values determined by the *J–V* curves.

**Table 1:** Best and Average (10–20 Devices for Each Concentration) Photovoltaic Parameters of Devices without and with AF32 with Different Concentrations

AF32 (mg/mL)	*V*_OC_ (mV)	*J*_SC_ (mA cm^–2^)	FF	PCE (%)
0.0	1101 (1085 ± 8.1)	24.11 (23.83 ± 0.18)	0.77 (0.76 ± 0.01)	20.3 (19.6 ± 0.3)
0.5	1109 (1097 ± 7.9)	24.24 (23.98 ± 0.16)	0.78 (0.77 ± 0.01)	20.9 (20.3 ± 0.3)
1.0	1142 (1132 ± 6.6)	24.52 (24.26 ± 0.16)	0.79 (0.78 ± 0.01)	22.1 (21.5 ± 0.3)
1.5	1147 (1139 ± 6.4)	24.57 (24.39 ± 0.12)	0.80 (0.79 ± 0.01)	22.6 (21.9 ± 0.2)
2.0	1124 (1101 ± 13.5)	24.16 (23.89 ± 0.14)	0.78 (0.77 ± 0.01)	21.2 (20.3 ± 0.4)
5.0	1093 (1073 ± 10.5)	22.34 (21.83 ± 0.32)	0.75 (0.74 ± 0.01)	18.4 (17.3 ± 0.6)

The charge recombination dynamics were further studied by measuring the light-intensity dependence of *V*_OC_ of relevant PSCs (Figure 5d). The slope of the control device was determined as 1.54 *kT/q*, where the slope indicates trap-assisted recombination. The slope was reduced to 1.26 *kT/q* when the interfacial layer AF32 was introduced between the perovskite layer and the HTL, indicating suppressed trap-assistant recombination which results in improved photovoltaic characteristics.

Essentially, the *V*_OC_ of devices correlates with the built-in potential (*V*_bi_), which is influenced by the population of photogenerated carriers during illumination. In Figure 5e, the Mott–Schottky (M–S) plots for the PSCs, utilizing both control and AF32-modified films, are presented. Notably, an increase of over 50 mV in the *V*_bi_ value was observed for the cell utilizing AF32, aligning consistently with the *V*_OC_ enhancement derived from the *J–V* scanning curves. This elevation likely stems from the suppression of nonradiative recombination, thereby maximizing the population of photogenerated carriers.^47^ Furthermore, electrical impedance spectroscopy (EIS) measurements were performed to further investigate the charge transport dynamics of the PSCs. The Nyquist plots of the control and modified PSCs under dark conditions at 1.0 V are represented in Figure 5f. It is clear that the series resistance (*R*_s_) values of the two devices are similar. However, notable variations are observed in the recombination resistance (*R*_rec_) and charge transfer resistance (*R*_ct_) values upon AF32 modification. Specifically, the *R*_rec_ value for the AF32-treated PSC increased to 16.0 kΩ, in contrast to that of the control PSC with a value of 13.2 kΩ. Conversely, the *R*_ct_ of the device modified by AF32 is lower (1.46 kΩ) than that of the control device (1.51 kΩ). These findings indicate a more efficient charge carrier transfer at the interface and suppressed interface recombination in the presence of the AF32 passivation molecule.^48,49^ This observation aligns well with the previously established link between interfacial defects, charge recombination kinetics, and overall device performance.^50^

**Figure 6:**
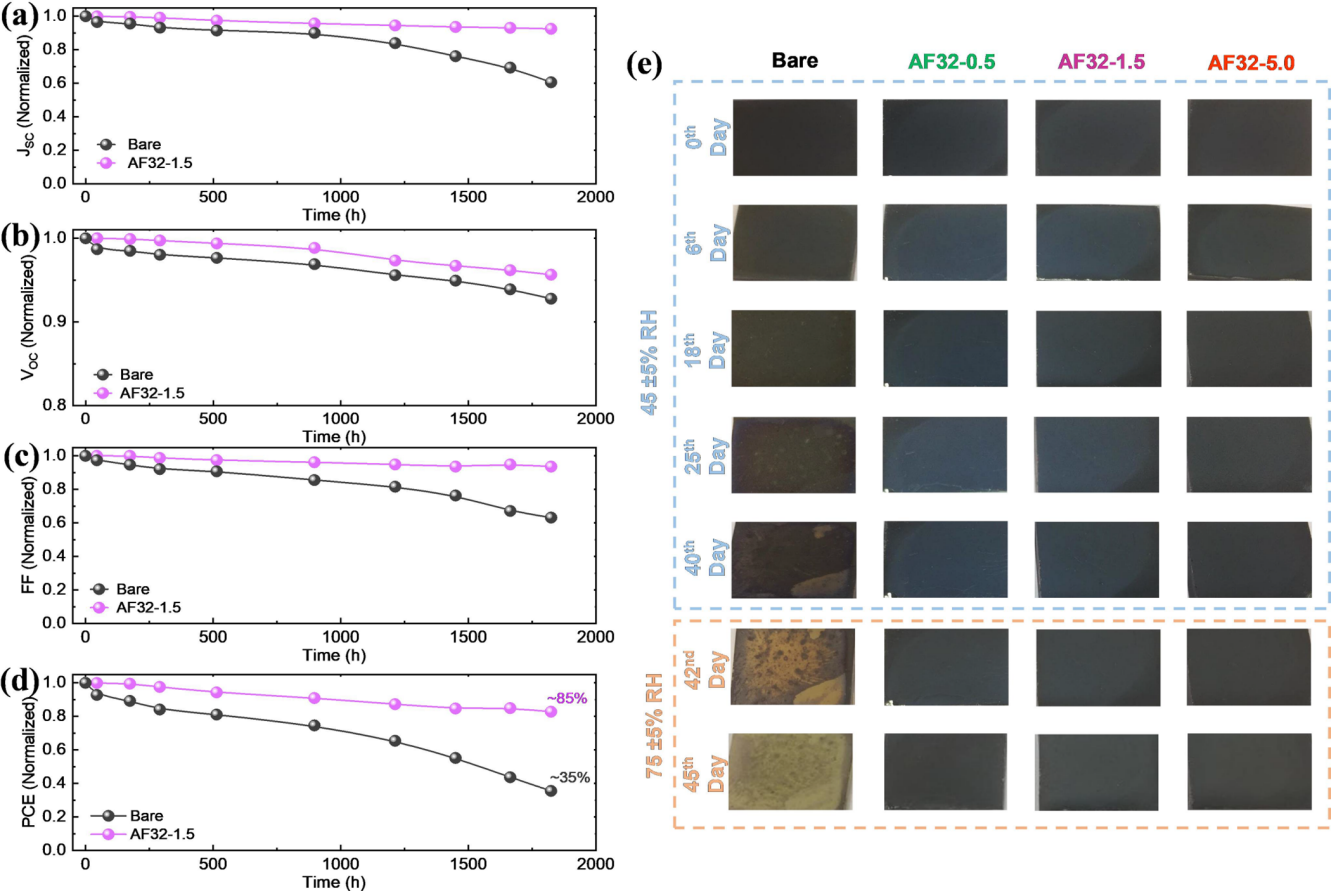
Photovoltaic stability results of devices without and with AF32 under ambient conditions (45 ± 5% RH, RT, and light soaking); (a) *J*_SC_, (b) *V*_OC_, (c) FF, and (d) PCE. (e) Time-dependent pictures of bare and AF32-passivated perovskite films under ambient conditions with different humidity levels (45 ± 5% to 75 ± 5% RH, RT, and light soaking).

Along with high efficiency, long-term stability is another key parameter for the commercial application of PSCs. All PV parameters for the PSCs with and without AF32 were investigated under ambient conditions (under radiation at room temperature and 45 ± 5% RH) (Figure 6a–d). The initial PCE values of the devices are 19.39 and 21.31% for control and AF32-modified devices, respectively. The device based on AF32 maintained 85% of its initial PCE over 1800 h, whereas the PCE of the control device was significantly reduced to 35% of its initial value under the same conditions. To exclusively investigate the impact of AF32 on the stability of the perovskite film, while excluding the influence of overlayers, time-dependent digital images of the control and AF32 treated perovskite films, aged under different RH from 45 ± 5 to 75 ± 5% for 45 days, were presented in Figure 6e. The color of the control perovskite film changed to yellow after 45 days which reflects the phase degradation to PbI_2_.^51^ On the other hand, the perovskite films passivated with different concentrations of AF32 showed no apparent change in color and remained black, indicating the defect passivation effect and higher hydrophobicity of perovskite film provided by the AF32 due to its long alkyl chains. It is widely recognized that moisture-induced deterioration occurs in perovskites. So, various alkyl chains have been incorporated into the AF32 molecule to impart some hydrophobic character to it. Water contact angle measurement studies were carried out for perovskite films with AF32 at different concentrations to understand the hydrophobic nature of different perovskite films. As demonstrated in Figure S10, the water contact angle increases with increasing AF32 concentration. The pristine perovskite film shows a contact angle of 51.1°, which increases to 84.7° when 5 mg/mL AF32 is deposited on the perovskite layer. This study indicates that the passivation with AF32 molecules makes the surface hydrophobic and prevents water from penetrating the perovskite layer, potentially enhancing the moisture stability of the entire cell.^52^

Top-view SEM analysis was performed for the films without and with AF32-1.5 after 30 and 45 days of aging under different relative humidity from 45 ± 5 to 75 ± 5%, respectively (Figures S11 and S12). As the humidity level increased, SEM images illustrated a rapid deterioration of the control perovskite film. In contrast, the passivated perovskite film maintained a stable surface morphology that remained largely unaffected, even under high RH. These findings demonstrate that AF32 offers stability to the perovskite film, particularly under challenging environmental conditions. UV–vis studies also validated the phase stability of the aged perovskite films modified by AF32 molecules. As shown in Figure S13, the passivated perovskite structure was retained for up to 35 days of aging under ambient conditions (RT, 45 ± 5% RH). In addition, XRD measurements were also conducted after 35 days of storage under ambient conditions (Figure S14). The highly intense PbI_2_ peak in the XRD pattern of the control perovskite film reveals the strong decomposition of perovskite to PbI_2_ whereas, the perovskite film with AF32 contains only the traces of PbI_2_ indicating the phase stability generated by passivation. In short, the passivated perovskite film exhibited remarkable resilience by maintaining a stable surface morphology, even under elevated RH levels. This resilience, attributed to the presence of AF32, highlights its capacity to provide stability to perovskite films, especially under challenging environmental conditions.

## Conclusions

To conclude, we designed and synthesized an organic molecule, effectively integrating it as a versatile interlayer within the PSCs. This molecular interlayer serves a dual purpose: it passivates surface defects inherent in the perovskite film and concurrently facilitates the efficient transport of holes. This innovative approach has yielded noteworthy advancements in both the performance and stability of PSC devices. We demonstrated that introducing a passivating layer, AF32, between the perovskite and HTL suppressed the deleterious nonradiative recombination and improved hole transportation/extraction. As a result of these advancements, the optimized modified device, configured as FTO/c-SnO_2_/perovskite/AF32/spiro-OMeTAD/Au has achieved a commendable PCE of 22.6%. This achievement is marked by negligible hysteresis effects and, perhaps more remarkably, by an exceptional long-term stability profile by retaining 85% of its initial PCE over 1800 h under ambient conditions. In summary, the integration of rational molecular design with strategic interfacial engineering holds promise as a potent strategy for ushering in a new era of efficient and enduring photovoltaic technology.

## Supporting Information Available

Synthetic scheme of AF32, synthetic procedure, spectroscopy (1H, 13C, and MALDI-TOF MS), and stability data of perovskite films with and without AF32 (PDF)
