# Supplementary material for: Molecularly Engineered Multifunctional Bridging Layer Derived from Dithiafulavene Capped Spiroxanthene for Stable and Efficient Perovskite Solar Cells
Source: ACS Appl Mater Interfaces. 2024 Mar 26;16(13):16213–23. doi: 10.1021/acsami.3c19619 (PMC10995899; doi:10.1021/acsami.3c19619)
Supplement: Supplementary file 1 — am3c19619_si_001.pdf [file am3c19619_si_001.pdf]

Supporting Information

Molecularly Engineered Multifunctional Bridging Layer Derived from Dithiafulavene Capped Spiroxanthene for Stable and Efficient Perovskite Solar Cells

Afzal Siddiqui,[a,b,#] Faranak Sadegh,[c,#] Kodali Phani Kumar,[a,b] Priksha Rana,[a] Pankaj Yadav[d,e], Daniel Prochowicz[f], Surya Prakash Singh,[a,b*] Seckin Akin,[c,g*]

[a] Department of Polymers and Functional Materials, CSIR-Indian Institute of Chemical Technology (IICT), Uppal Road, Tarnaka, Hyderabad, 500007, India. E-mail: spsingh@iict.res.in
[b] Academy of Scientific and Innovative Research (AcSIR), Ghaziabad, 201002, Uttar Pradesh, India.
[c] Laboratory of Advanced Materials & Photovoltaics (LAMPs), Necmettin Erbakan University, 42090, Konya, Turkey.
[d] Department of Solar Energy, School of Energy Technology, Pandit Deendayal Energy University, Gandhinagar, 382007, Gujarat, India.
[e] Department of Physics, School of Energy Technology, Pandit Deendayal Energy University, Gandhinagar, 382007, Gujarat, India.
[f] Institute of Physical Chemistry, Polish Academy of Sciences, 01-224, Warsaw, Poland.
[g] Department of Metallurgical and Materials Engineering, Necmettin Erbakan University, 42090, Konya, Turkey.
E-mail: seckinakin@erbakan.edu.tr

[#] These authors contributed equally to this work.

Table of contents

1. Synthetic scheme of AF32……………………………………………………………S3

2. Synthetic Procedure……………………………………………………………….S4

2.1 Synthesis of SFX………………………………………………………………..S4

2.2 Synthesis of SFX-EH……………………………………………………………...S4

2.3 Synthesis of SFX-Bpin……………………………………………………………S4

2.4 Synthesis of PTZ-CHO………………………………………………………....S5

2.5 Synthesis of SFX-PTZ……………………………………………………………..S5

2.6 Synthesis of AF32…………………………………………………………………S6

3. Spectroscopy (^{1}H, ^{13}C and MALDI-TOF MS) ………………………………………..S7-S10

4. Absorption spectroscopy and Cyclic voltammetry of AF32…………………………....S11

5. SEM images and grain size histograms of perovskite films with AF32……….…..... S12-S13

6. AFM images of perovskite films with AF32…………………………………...…S14

7. UV-vis absorption spectroscopy of perovskite films with AF32………………………S15

8. XPS spectra………………………………………………………………..….…...S16

9. V_{OC}, J_{SC}, FF and PCE data……………………………………………………… S17-S18

10. Water contact angle images……………………………………………………...…S19

11. Stability data of perovskite films with and without AF32…………………………. S20-S21

12. Tables (Table S1 and Table S2)……………………………………………………S22

13. References…………………………………………………………………..……S23

1. Synthetic Scheme

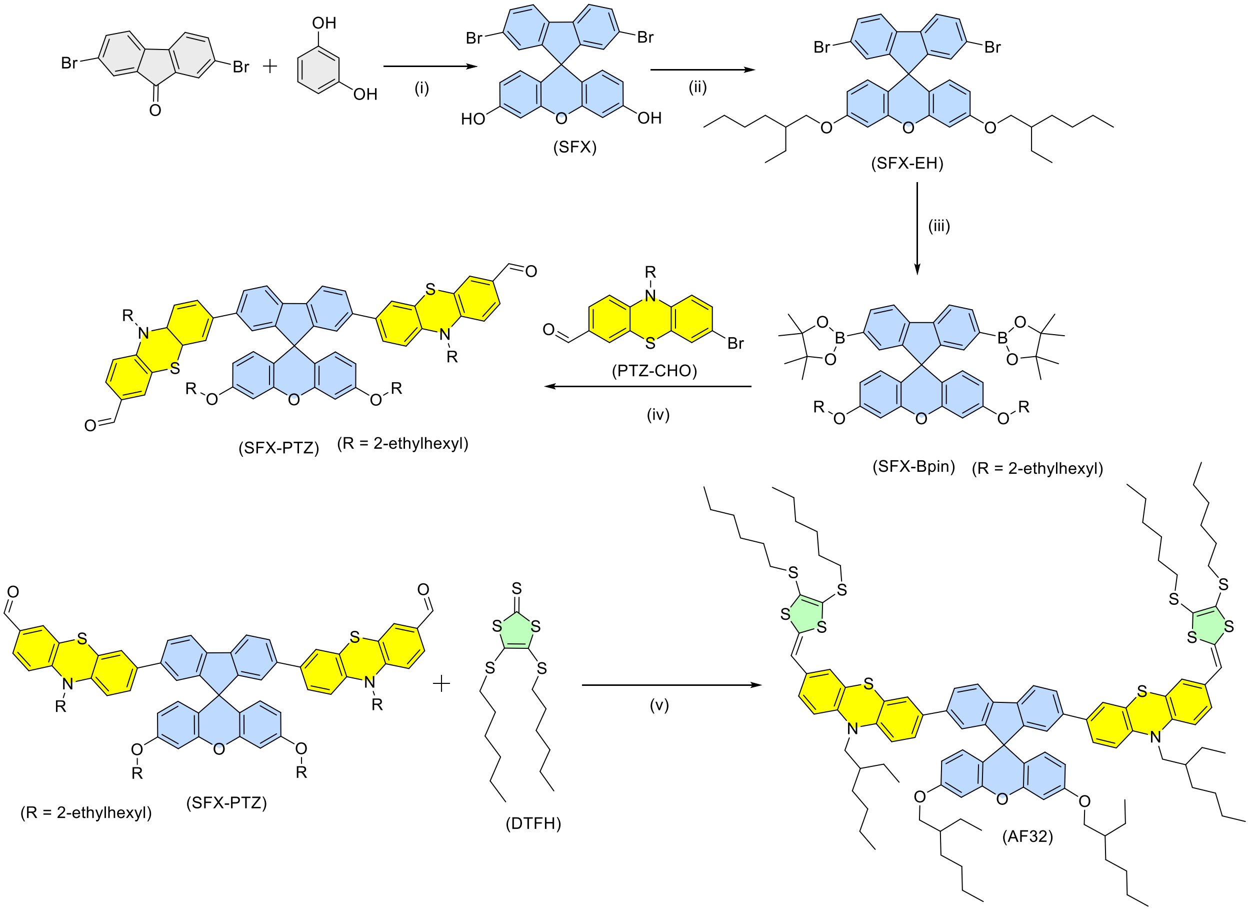

Fig. S1: Reagents and conditions: (i) p-toluenesulfonic acid, toluene, 110°C, 24h, 67%, **(ii)** 2-ethylhexyl bromide, K_2CO_3, DMF, 12h, reflux, 63%, **(iii)** bis(pinacolato)diboron, CH_3COOK, Pd(dppf)Cl_2, 1,4-dioxane, 24h, 110°C, 75%, **(iv)** Pd $(PPh_3)_4$, K_2CO_3, THF, 24h, 65°C, 70%, **(v)** $CHCl_3$, triethyl phosphite, 90°C, 12h, 65%.

2. Synthetic procedure:

2.1 Synthesis of SFX:

A 100 mL round bottom flask was charged with 2,7 dibrom-9- fluorenone (1.750 g, 5.177 mmol), resorcinol (2.280 g, 20.710 mmol), p-TsOH (0.100 g,0.051 mmol) and toluene (40 mL). The mixture was refluxed for 12h and then cooled to room temperature. After completion of the reaction, water (100 mL) was added and the mixture was stirred at room temperature for 0.5h. The yellow colour precipitated from the reaction mixture was isolated by filtration and washed with water several times. The crude was dissolved in ethyl alcohol (100 mL) and the organic solvent was concentrated by vacuum evaporation and purified by column chromatography to give the white solid (1.90g, 67% yield). The characterization is well-matched with the reported one[1].

2.2 Synthesis of SFX-EH:

Compound 2 was prepared as per the literature.[2] SFX (1.8g, 3.45 mmol, 1equiv), 2-ethylhexyl bromide (2 mL, 10.35 mmol, 3 equiv), K_2CO_3 (0.773g, 13.79 mmol, 4equiv) and DMSO (10 mL) were added to a single-necked round bottom flask. The mixture was refluxed for 12h and then cooled to room temperature. The mixture was extracted with ethyl acetate. The organic layer was dried with sodium sulphate, and evaporated under vacuum and the residue was further purified with column chromatography to give a colourless solid.

2.3 Synthesis of SFX-Bpin:

To a dry round bottom flask (100 mL) the compound 2 (0.912g, 1.22 mmol, 1equiv), Bis(pinacolato)diboron (0.932g, 3.672 mmol, 3equiv), potassium acetate (0.480g, 4.896 mmol, 4equiv) were added and dissolved in 1,4-dioxane (40 mL) under the nitrogen atmosphere. The mixture was degassed for 30 min. After that, we added Pd(dppf)Cl2 catalyst (10 mol%) and then the mixture was refluxed for 24h at 110°C. The reaction mixture was cooled to room temperature, filtered and washed with DCM several times. The reaction mixture was extracted with dichloromethane. The organic layer was dried over sodium sulphate and the solvent was evaporated under vacuum. The residue was further purified by column chromatography to give the white solid (1.15g, 92%). **^{1}H NMR (400 MHz, CDCl3 δ (ppm)** 7.85 – 7.72 (m, 4H), 7.54 – 7.46 (m, 2H), 6.67 (t, J = 18.5 Hz, 2H), 6.38 – 6.23 (m, 2H), 6.20 (t, J = 6.9 Hz, 2H), 3.91 – 3.67 (m, 4H), 1.69 (dd, J = 12.1, 6.0 Hz, 2H), 1.53 – 1.34 (m, 10H), 1.33 – 1.24 (m, 34H), 0.94 – 0.88 (m,

12H). **^{13}C NMR (101 MHz, CDCl3) δ (ppm)** 159.02, 159.02, 155.95, 151.91, 142.24, 131.96, 129.26, 119.45, 115.84, 111.06, 101.55, 83.76, 70.56, 53.22 , 39.35, 30.53, 29.73, 29.09, 24.89, 23.87, 23.09, 14.12, 11.14.

2.4 Synthesis of PTZ-CHO:

PTZ-CHO was synthesized in two steps [3]

Step-1: Dry phosphorus oxychloride (11.9 mL, 128.4 mmol) was added dropwise very slowly to the dry N, N-dimethylformamide (DMF) (8.0 mL, 102.7 mmol) at 0 ^{0}C in a 100 ml two neck round bottom flask under nitrogen atmosphere with stirring. After the formation of a white solid, 10-(2-ethylhexyl)-10H-phenothiazine (8.0 g, 25.6 mmol) was dissolved in 50 mL of dry 1,2-dichloroethane (DCE) was added dropwise for 0.5 h to the reaction mixture. The reaction was heated to reflux for 24 h. After cooling to room temperature diluted sodium hydroxide solution was added and extracted with ethyl acetate (200 ml × 3). The organic layer was dried with anhydrous Na_2SO_4 and then the solvent was removed using a rotatory evaporator. The residue was purified by silica gel column chromatography using ethyl acetate and hexane as the eluent to yield phenothiazine aldehyde as a yellow liquid. Yield (7.5g, 86.3%).

Step-2: In a round bottom dry flask (100 mL) phenothiazine aldehyde (0.930 g, 2.74 mmol) was dissolved in acetone (20 mL) and NBS (0.488 g, 2.74 mmol) was added portionwise up to 2h. The reaction was stirred for 6h at room temperature under dark conditions. After completion of the reaction, the solvent was evaporated under vacuum. The residue was further purified by column chromatography to give the yellow solid compound **4** (0.850 g, 75% yield). The characterization is matched with the reported literature.

2.5 Synthesis of SFX-PTZ:

compound 3 (0.500g, 0.595 mmol, 1equiv), 7-bromo-10-(2-ethylhexyl)-10H-phenothiazine-3-carbaldehyde 4 (0.546g, 1.309mmol, 2.2 equiv), K_2CO_3(0.246g, 1.7 mmol, 3 equiv) were dissolved in THF/ H_2O (2:1) mixture (20 mL) in 100 mL round bottom flask and degassed for 30 min with argon gas. After that $Pd(PPh3)_4$ (1.0 mol %) was added in the presence of Ar gas. The reaction mixture was refluxed for 24 h at 70°C. The completion of the reaction was monitored using TLC. Water was added to the reaction mixture and extraction was carried out with dichloromethane. The organic layer was dried over Na_2SO_4. The solvent was evaporated under

reduced pressure. The product was purified by column chromatography using 40% DCM-hexane as eluent. The light green solid (fluorescence) product obtained (0.530g yield 70 %). **^{1}H NMR (300 MHz, $CDCl_3$) δ (ppm)** 9.79 (s, 2H), 7.81 (d, *J* = 7.9 Hz, 2H), 7.64 (d, *J* = 8.3 Hz, 2H), 7.60 (d, *J* = 1.6 Hz, 2H), 7.55 (d, *J* = 6.9 Hz, 2H), 7.31 – 7.26 (m, 6H), 6.89 (dd, *J* = 18.1, 8.4 Hz, 4H), 6.75 (t, *J* = 4.2 Hz, 2H), 6.34 (d, *J* = 8.1 Hz, 4H), 3.82 (d, *J* = 5.5 Hz, 4H), 3.77 (d, *J* = 6.9 Hz, 4H), 1.90 (d, *J* = 5.5 Hz, 2H), 1.76 – 1.58 (m, 8H), 1.35 (ddd, *J* = 18.3, 12.0, 4.9 Hz, 34H), 0.92 – 0.84 (m, 24H). **^{13}C NMR (101 MHz, $CDCl_3$) δ (ppm)** 190.12, 159.29, 156.75, 152.04, 151.36, 143.04, 139.72, 138.40, 136.47, 131.14, 130.00, 129.77, 128.84, 126.16, 125.97, 125.78, 125.26, 123.66, 120.84, 120.29, 116.67, 116.25, 115.46, 111.18, 101.72, 70.58, 53.61, 51.50, 39.34, 38.89, 33.24, 31.98, 30.60, 30.53, 30.22, 29.76, 29.09, 28.55, 26.78, 25.93, 24.91, 23.87, 23.08, 22.75, 14.14, 14.04, 11.15, 10.94, 10.46.

2.6 Synthesis of AF32: A dry 25 mL round bottom flask charged with Compound 5 (0.200g, 0.158 mmol, 1 equiv), and 4,5-bis(hexylthio)-1,3-dithiole-2-thione (DTF) (0.133g, 0.396 mmol, 2.5 equiv) were dissolved in toluene (20 mL). Under inert conditions, the reaction mixture refluxes for 30 minutes. Then triethyl phosphite was added to the mixture dropwise with a syringe and refluxed for 5 hours. After completion of the reaction, the solvent was evaporated and then extracted from the organic layer with DCM solvent and dried over anhydrous sodium sulphate. The product was further purified by column chromatography. The dark green-coloured solid product formed (0.150g yield 50%). **^{1}H NMR (400 MHz, $CDCl_3$) δ (ppm)** 7.79 (d, *J* = 7.9 Hz, 2H), 7.56 – 7.50 (m, 2H), 7.31 – 7.26 (m, 6H), 6.98 (d, *J* = 8.1 Hz, 4H), 6.82 (d, *J* = 7.8 Hz, 4H), 6.74 (d, *J* = 1.7 Hz, 2H), 6.39 – 6.33 (m, 4H), 6.31 (s, 2H), 4.24 – 4.05 (m, 8H), 3.82 (d, *J* = 5.7 Hz, 4H), 3.70 (d, *J* = 6.9 Hz, 4H), 2.81 (t, *J* = 6.9 Hz, 8H), 1.89 (d, *J* = 5.6 Hz, 2H), 1.72 – 1.60 (m, 4H), 1.38 (ddd, *J* = 10.5, 7.9, 3.9 Hz, 12H), 1.31 (dd, *J* = 9.9, 1.6 Hz, 40H), 0.88 (tt, *J* = 18.9, 7.4 Hz, 36H). **^{13}C NMR (101 MHz, $CDCl_3$) δ (ppm)** 159.23, 156.65, 152.04, 144.56, 143.33, 138.20, 135.25, 131.03, 130.14, 128.83, 127.49, 125.80, 125.66, 125.48, 124.56, 123.58, 122.81, 116.42, 115.88, 115.64, 113.50, 111.13, 101.74, 70.59, 63.73, 53.60, 51.07, 39.35, 36.17, 36.05, 35.96, 31.38, 30.73, 30.54, 29.84, 29.70, 29.09, 28.62, 28.29, 23.98, 23.88, 23.08, 22.58, 14.12, 14.06, 11.14. MALDI-TOF calculated for $C_{113}H_{146}N_2O_3S_{10}$: 1898.85 found $[M]^{+1}$ 1899.12

3. Spectroscopy:

^{1}H NMR of SFX-Bpin:

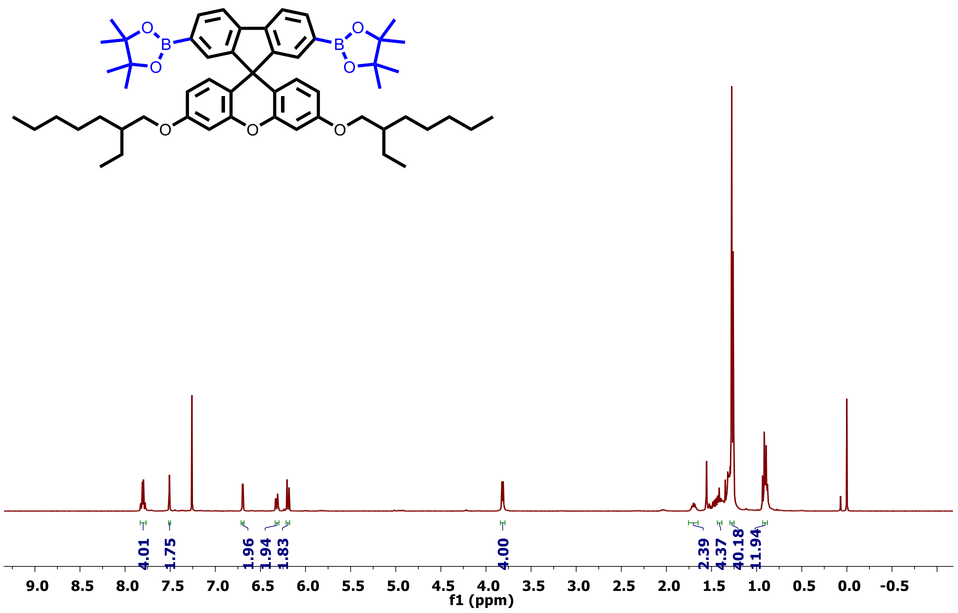

^{13}C NMR of SFX-Bpin:

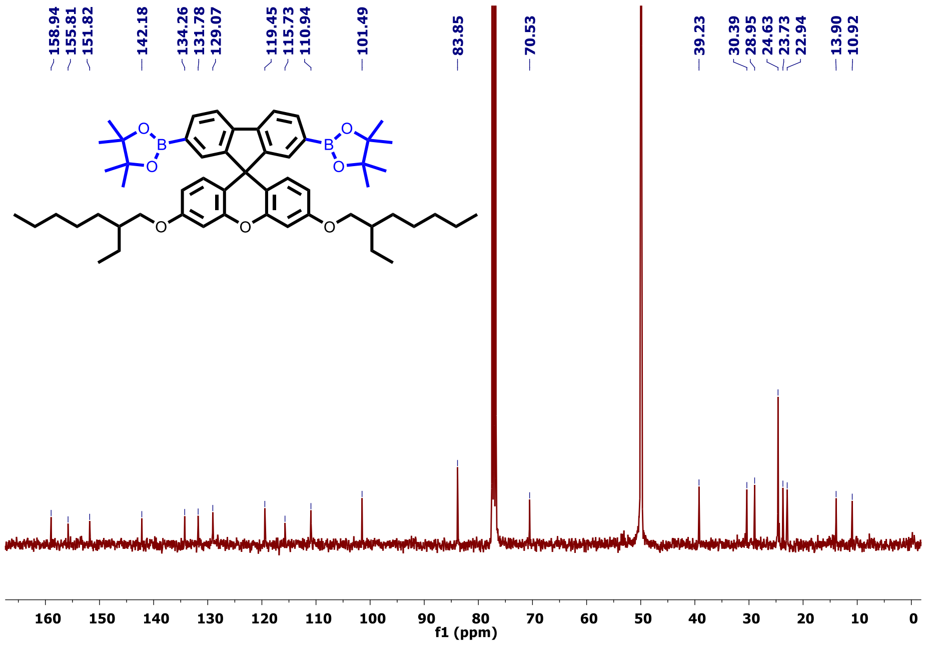

^{1}H NMR of SFX-PTZ:

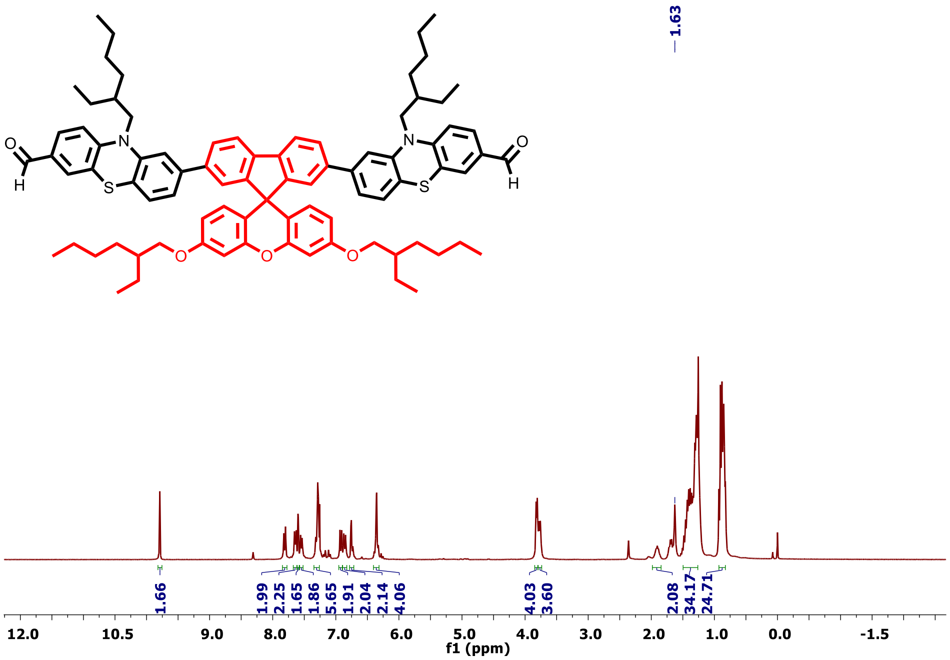

^{13}C NMR of SFX-PTZ:

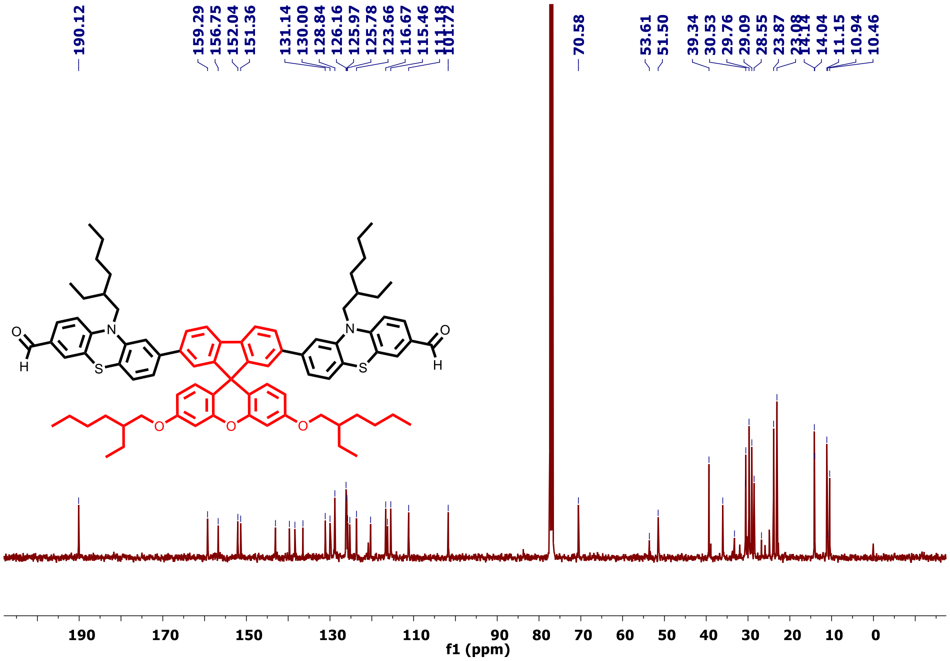

^{1}H NMR of AF32

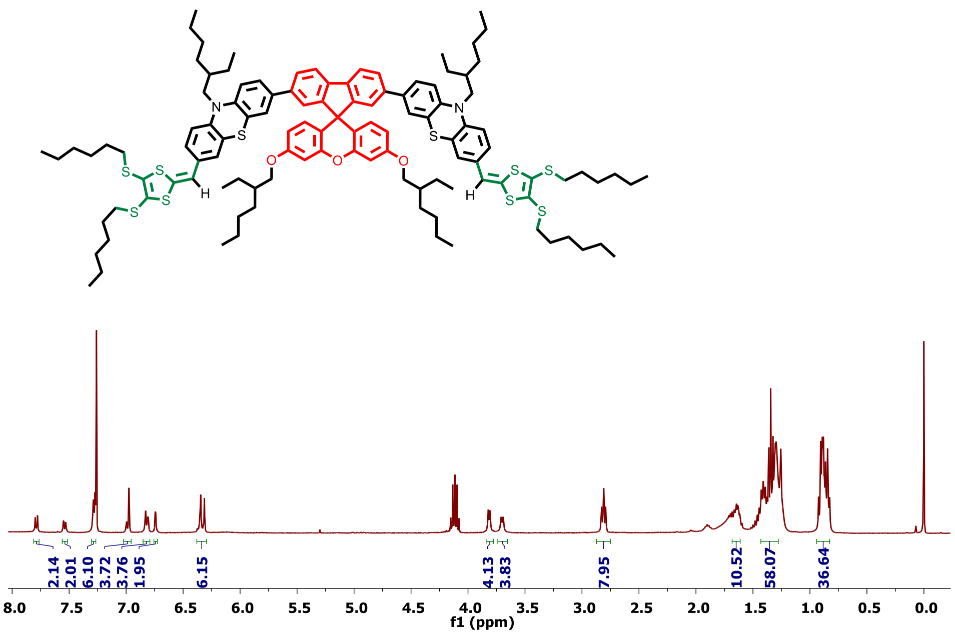

^{13}C NMR of AF32

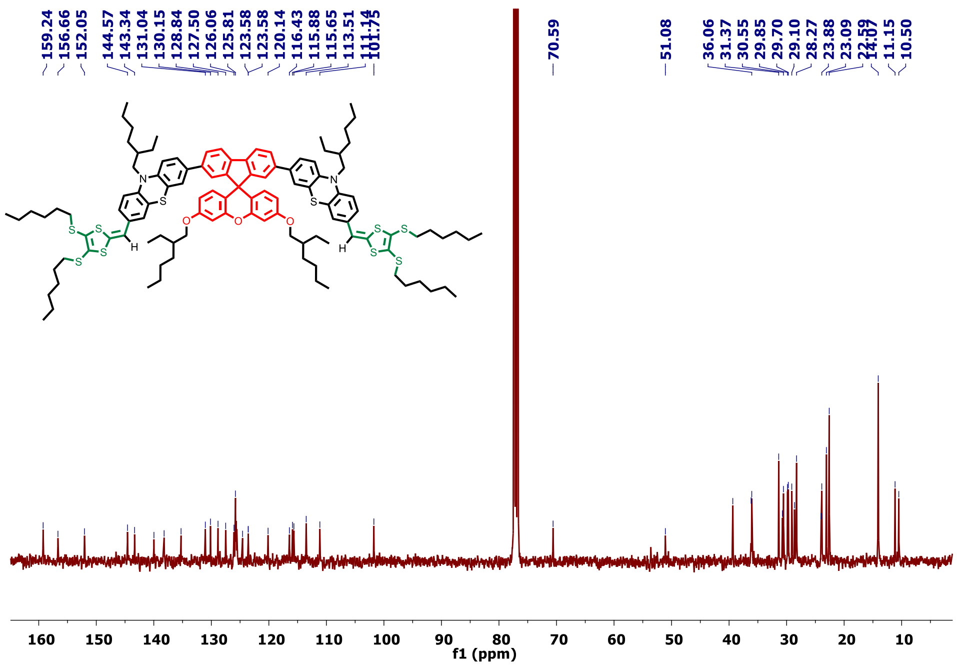

MALDI-TOF spectra of AF32:

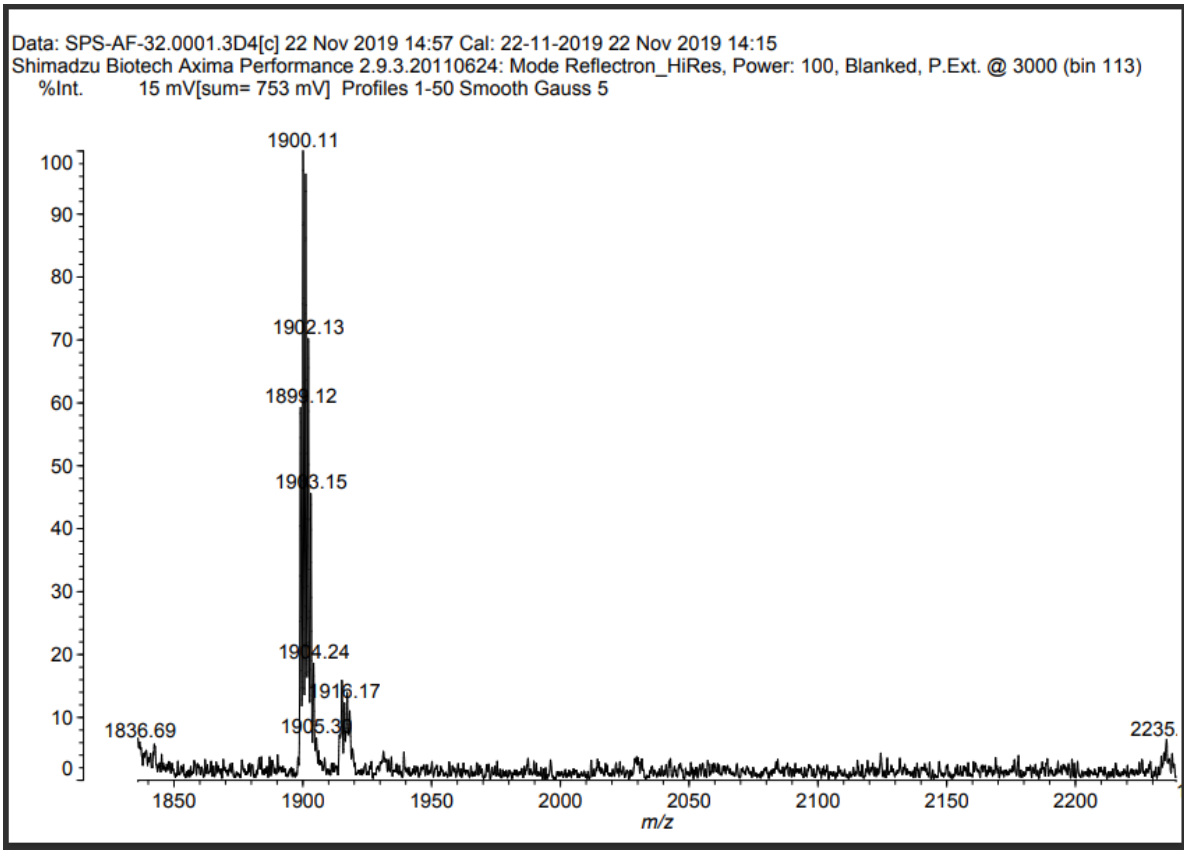

4. Absorption spectroscopy and cyclic voltammetry of AF32:

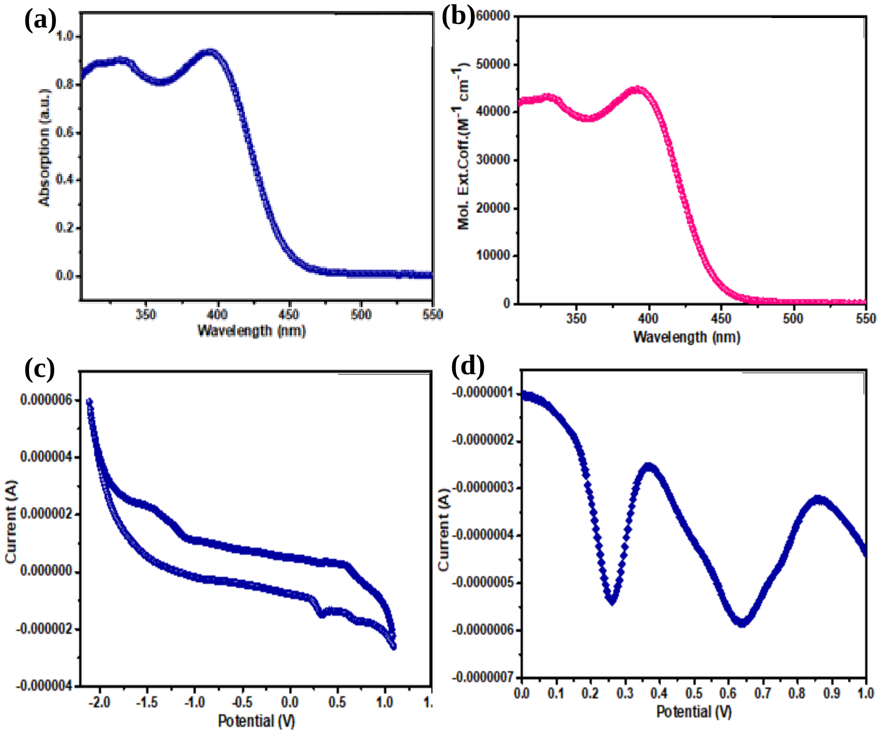

Fig. S2: a) Normalized UV-visible absorption and (b) Molar extinction coefficient spectra of AF32 in dichloromethane solvent. (c) The cyclic voltammetry and (d) differential pulse voltammetry curves of AF32 in dichloromethane solvent.

5. SEM images and grain size histograms of perovskite films with AF32:

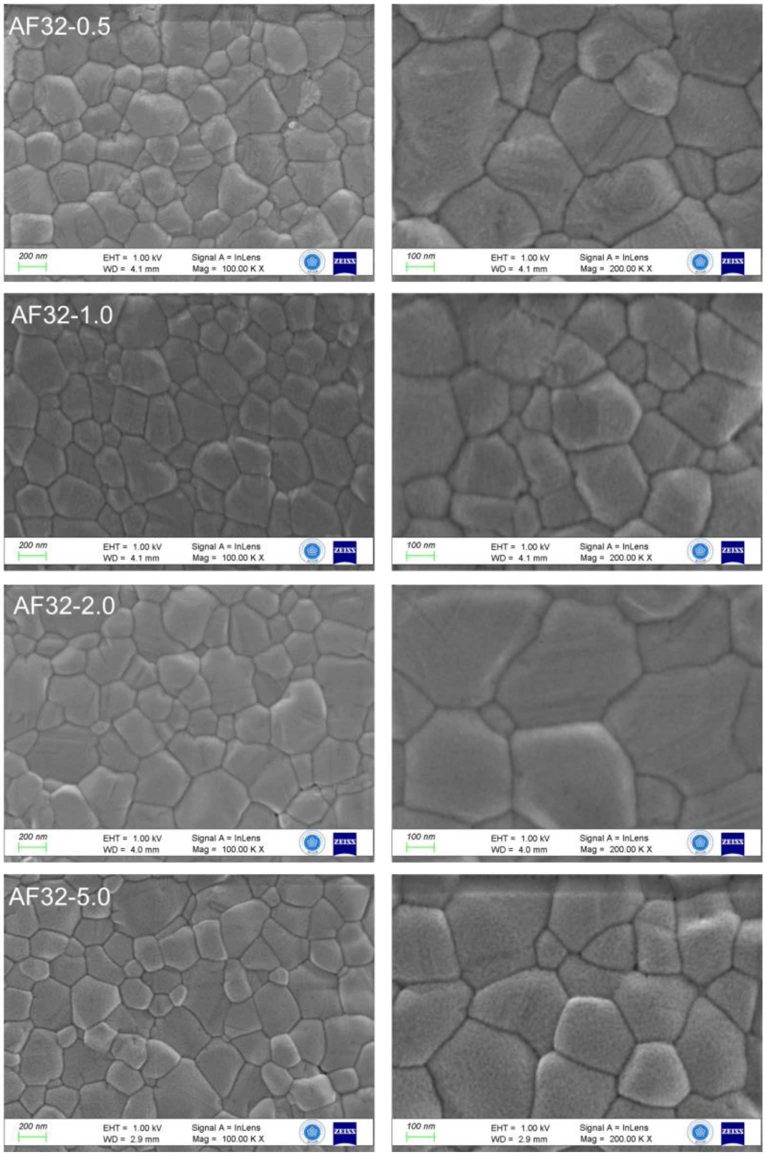

Fig. S3: Top-view SEM micrographs at different magnifications of perovskite films employing AF32 with different concentrations.

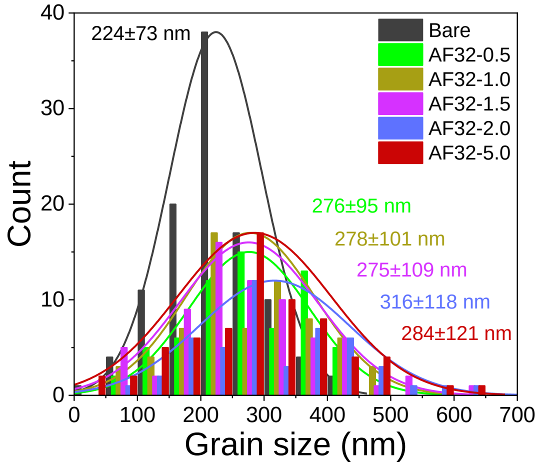

Fig. S4: Grain size histograms obtained from top-view SEM micrographs.

6. AFM images of perovskite films with AF32:

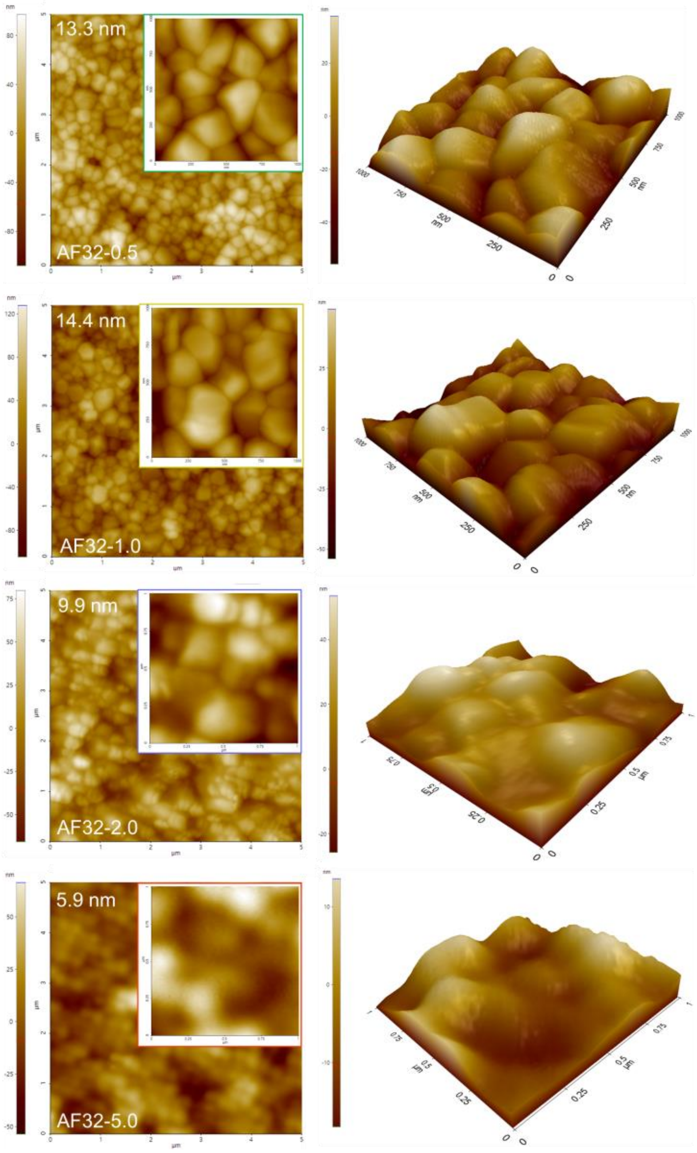

Fig. S5: AFM images of perovskite films with AF32 with different concentrations.

7. UV-vis absorption spectroscopy of perovskite films without and with AF32:

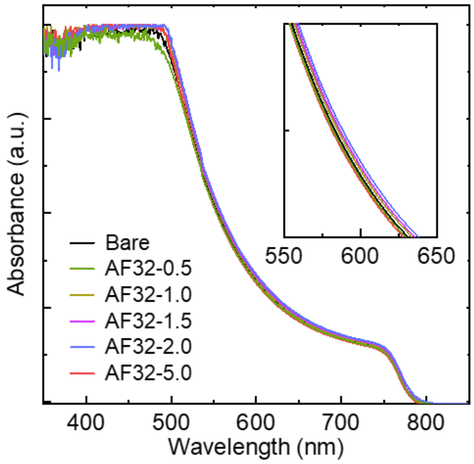

Fig. S6: UV-visible absorption spectra of perovskite films without and with AF32 with different concentrations.

8. XPS spectra:

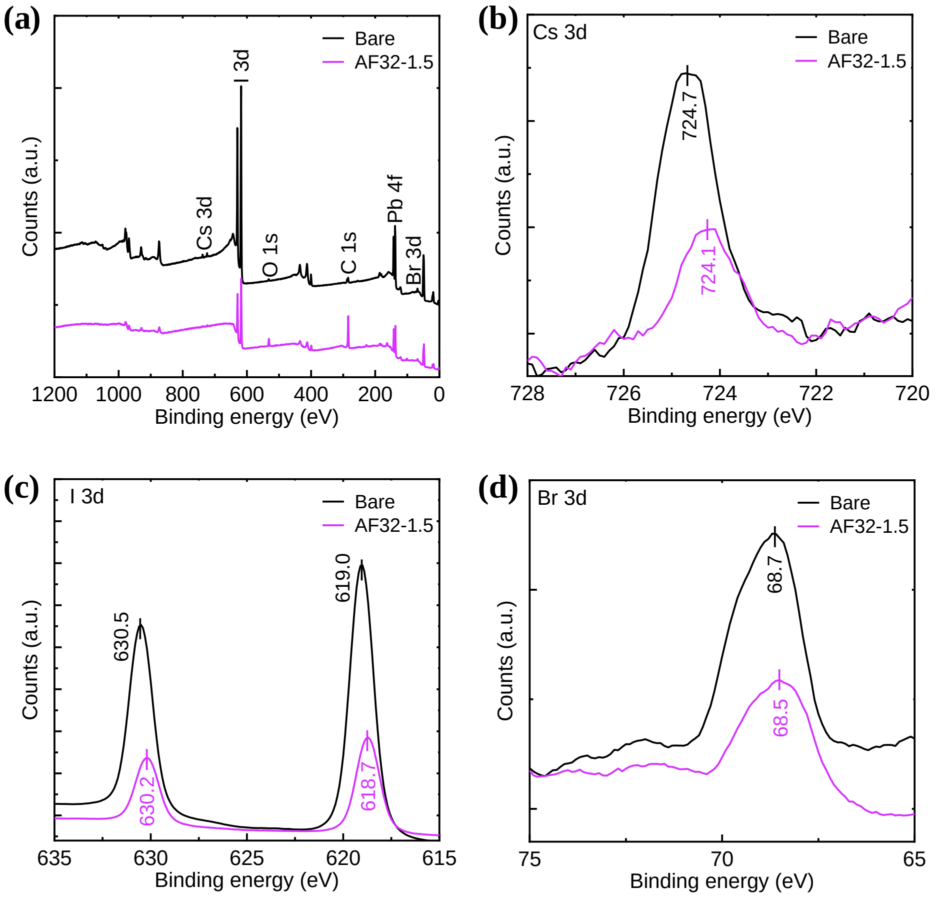

Fig. S7: a) Survey and high-resolution (b) Cs 3d, (c) I 3d, (d) Br 3d core level XPS spectra of perovskite films with and without AF32.

9. V_{OC}, J_{SC}, FF and PCE data:

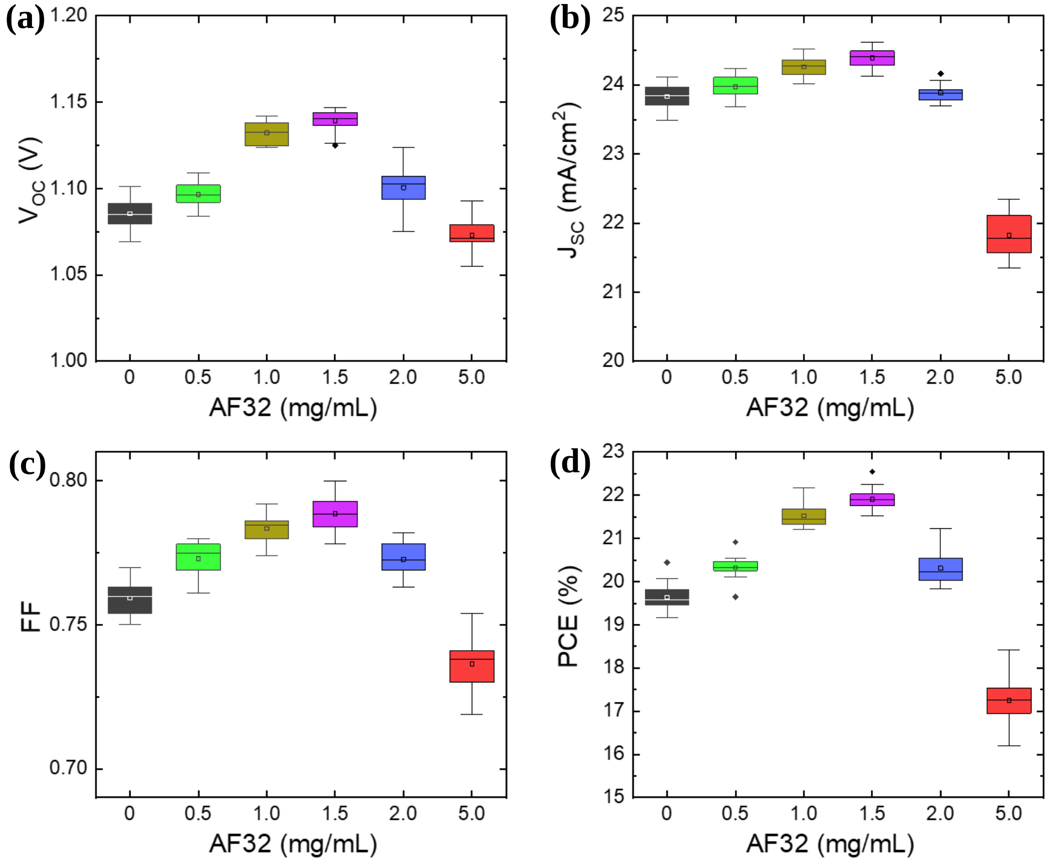

Fig. S8: Photovoltaic parameters (a) V_{OC}, (b) J_{SC}, (c) FF, and (d) PCE of PSCs without and with AF32 with different concentrations.

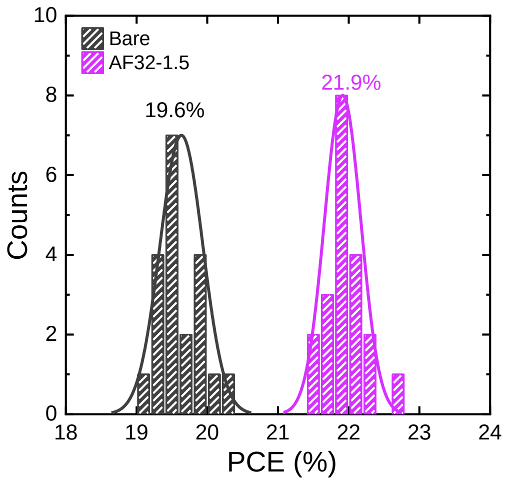

Fig. S9: PCE statistics for over 10 to 20 devices with and without AF32 interlayer.

10. Water contact angle images:

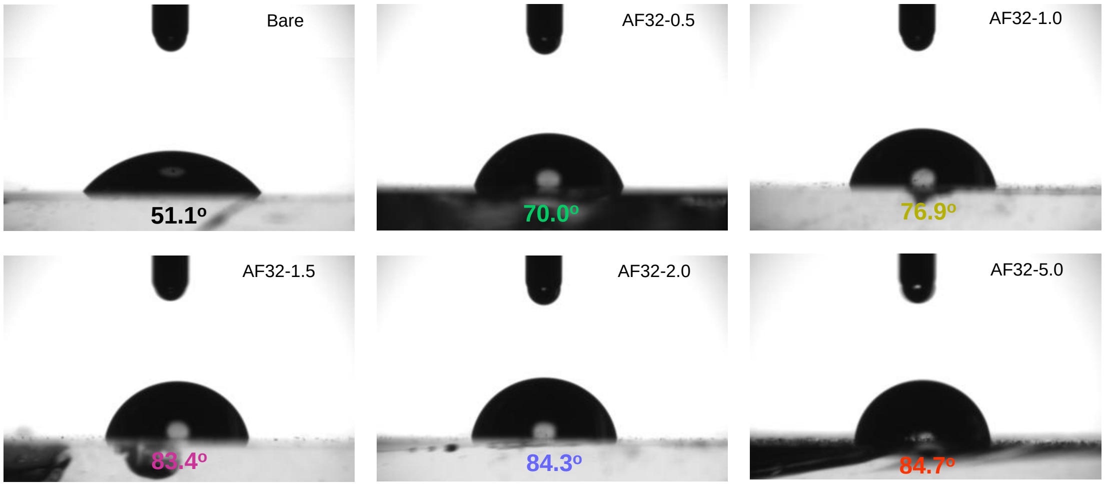

Fig. S10: Water contact angle of perovskite films without and with different concentrations of AF32.

11. Stability data of perovskite films with and without AF32:

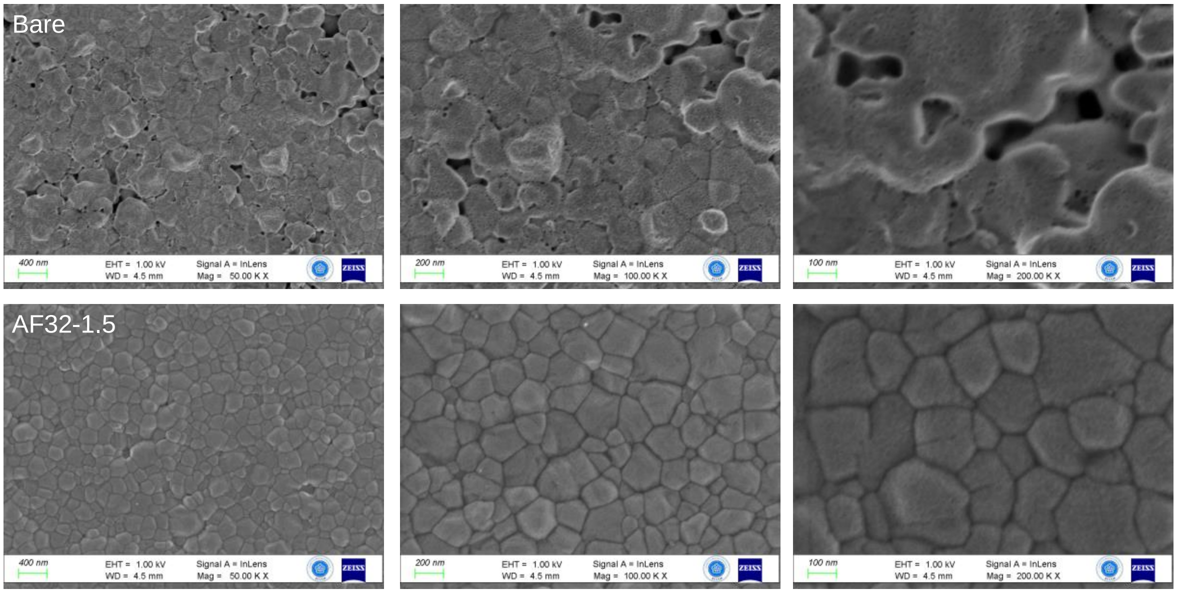

Fig. S11: Top-view SEM images at different magnifications of perovskite films without and with AF32 after 30 days aged under ambient conditions (RT, 45±5% RH).

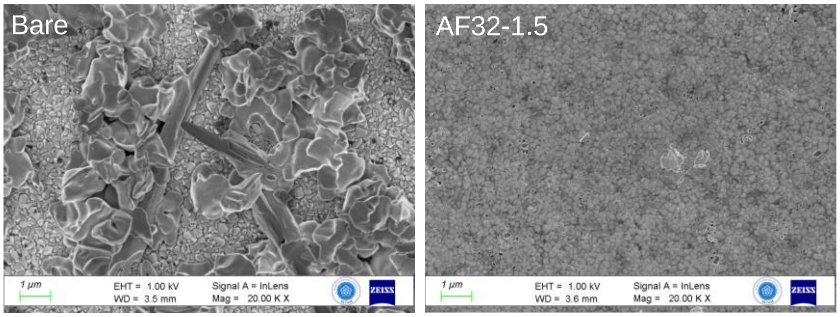

Fig. S12: Top-view SEM images of perovskite films without and with AF32 after 45 days aging under RT and different RH (45±5% to 75±5%).

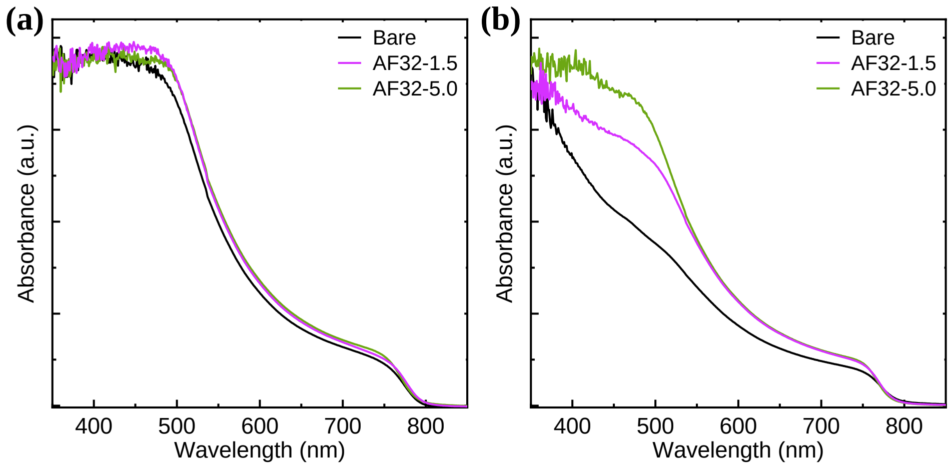

Fig. S13: Time-dependent absorbance spectra of bare and AF32 treated perovskite films after (a) 10 and (b) 35 days of ageing under ambient conditions (RT, 45±5% RH).

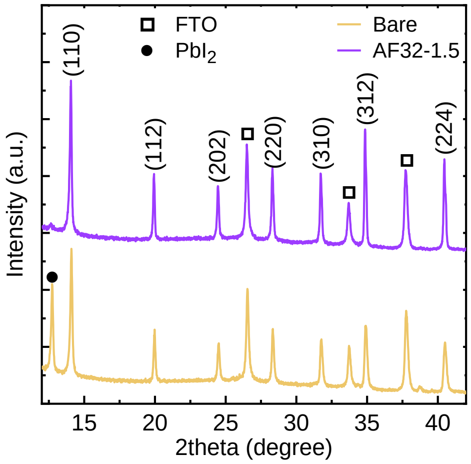

Fig. S14: Time-dependent XRD patterns of bare and AF32 passivated perovskite films after 35 days aged under ambient conditions (RT, 45 ±5% RH).

12. Tables:

Table S1: Optical and electrochemical properties of Spiro-xanthene-based AF32.

**Material**	**λ_{max1} (nm)**	**λ_{max2} (nm) (ε /M^{-1} cm^{-1})**	**E_{0-0} (eV)**	**E_{LUMO} (eV)**	**E_{HOMO} (eV)**
AF32	330	394 (44730)	2.73	-2.75	-5.48

Table S2: Summary of the carrier lifetime estimated from fitting TRPL spectra.

**Samples**	**t_1 (ns)**	**A_1**	**t_2 (ns)**	**A_2**	**t_{ave} (ns)**
Perovskite/ Spiro-OMeTAD	28.2	0.88	107.6	0.12	55.4
Perovskite/AF32-1.5/ Spiro-OMeTAD	4.9	0.78	40.2	0.22	29.6

13. References

1. Bo Xu, Zonglong Zhu, Jinbao Zhang, Hongbin Liu, Chu-Chen Chueh, Xiaosong Li, Alex K.-Y. Jen,Adv. *Energy Mater*. **2017**, 1700683.
2. B. Yadagiri, T.H. Chowdhury, Y. He, R. Kaneko, A. Islam and S.P. Singh, *Mater. Chem. Front.*, 2021, 5, 7276–7285
3. Yadagiri, B.; Narayanaswamy, K.; Srinivasa Rao, R.; Bagui, A.; Datt, R.;Gupta V.; Singh S. P., *ACS Omega*., **2018**, 3, 13365–13373.
